# Food-Derived β-Carboline Alkaloids Ameliorate Lipid Droplet Accumulation in Human Hepatocytes

**DOI:** 10.3390/ph15050578

**Published:** 2022-05-05

**Authors:** Dya Fita Dibwe, Saki Oba, Nire Takeishi, Toshihiro Sakurai, Takayuki Tsukui, Hitoshi Chiba, Shu-Ping Hui

**Affiliations:** 1 Faculty of Health Sciences, Hokkaido University, Kita-12, Nishi-5, Kita-Ku, Sapporo 060-0812, Japan; dibwedf@hs.hokudai.ac.jp (D.F.D.); sakura@hs.hokudai.ac.jp (T.S.); 2 Graduate School of Health Sciences, Hokkaido University, Kita-12, Nishi-5, Kita-Ku, Sapporo 060-0812, Japan; oba.saki.x0@elms.hokudai.ac.jp (S.O.); takeishi.nire.m0@elms.hokudai.ac.jp (N.T.); 3 Department of Nutrition, Sapporo University of Health Sciences, Nakanuma Nishi-4-3-1-15, Higashi-Ku, Sapporo 007-0894, Japan; tsukui@sapporo-hokeniryou-u.ac.jp (T.T.); chiba-h@sapporo-hokeniryou-u.ac.jp (H.C.)

**Keywords:** functional foods, *Crassostrea gigas*, β-carboline alkaloids, bioactive compounds, lipid droplet accumulation inhibition, lipidomics, neutral lipids, triacylglycerols

## Abstract

Lipid droplet accumulation (LDA) in hepatocytes is the initial stage of nonalcoholic fatty liver disease (NAFLD). In the search for natural compounds for the prevention of NAFLD, a series of β-carboline alkaloid derivatives, inspired by flazin and its derivative, newly identified in *Crassostrea gigas* Thunberg. extracts, were examined for LDA inhibition (LDAI) activity in oleic acid–loaded hepatocytes (HepG2). Eight compounds with a piperidine or pyridine C-ring were chemically synthesized (**1**–**8**). Among them, compounds **2** and **4** (flazin) with a carboxy group at C-3 and furfuryl alcohol moiety at C-1 showed low cytotoxicity and they exhibited significant LDAI activity. Compound **2** with piperidine C-ring was identified for the first time in *C. gigas* extract, and ameliorated the lipid accumulation with the LDAI value of 25.4%. Active compounds **2** and **4** significantly inhibited triacylglycerol species accumulation in cells. These compounds upregulated *ATGL* and downregulated *SREBP1*, *FASN*, and *SCD1* genes, suggesting that they activated lipolysis and suppressed lipogenesis, respectively. These results suggest that β-carboline alkaloids, especially compounds **2** and **4**, might be potentially useful for preventing NAFLD.

## 1. Introduction

Nonalcoholic fatty liver disease (NAFLD) is the most prevalent chronic liver disease worldwide. It is associated with obesity, insulin resistance, type 2 diabetes mellitus, hypertension, dyslipidemia, and metabolic syndrome. The global epidemics of obesity and type 2 diabetes mellitus have led to a very high prevalence of NAFLD in the civilized Western and Eastern countries, such as the US, EU, China, and Japan, in the last two decades. Nonalcoholic steatohepatitis (NASH) is a progressive form of NAFLD. It can cause cirrhosis, hepatocellular carcinoma, and hepatic failure [1,2,3,4,5]. Currently, there are no approved pharmacotherapies specific to NASH [6]. Therefore, a strategy for preventing and managing NAFLD/NASH is urgently required. An increasing number of studies report an association between excessive intracellular lipid droplet accumulation (LDA) and obesity, diabetes, and other metabolic disorders [7,8,9,10]. Hepatic LDA is thought to initiate the early stages of NAFLD [11]. Agents that can reduce LDA in hepatocytes/the liver represent promising candidates for preventing and managing obesity-associated NAFLD [3,12,13,14,15,16,17]. Food-derived natural bioactive products with LDAI activity in hepatocytes are potential candidates for this.

β-Carboline alkaloids are a vast group of natural and synthetic indole alkaloids. Some are widely distributed in nature, e.g., in various plants and foodstuffs [18]. A broad spectrum of their pharmacological properties has been reported. Such reports have mentioned sedative, anxiolytic, hypnotic, anticonvulsant, antitumor, antiviral, antiparasitic, and antimicrobial activity [18,19,20]. We have reported that a β-carboline-derived alkaloid, flazin, from a functional food extract (Pacific oyster, *Crassostrea gigas* Thunberg) protects cultured human hepatocytes (C3A) from oxidative damage by a pro-oxidant, 2,2′-azobis-2-methyl-propanimidamide dihydrochloride (AAPH), without showing any significant cytotoxicity. Flazin activated the nuclear factor erythroid 2-related factor 2 (Nrf2) and exerted a strong antioxidant action by activating multiple Nrf2-dependent antioxidant enzymes. A similar Nrf2-dependent antioxidant action in hepatocytes was observed in 3,5-dihydroxy-4-methoxybenzyl alcohol (DHMBA) derived from Pacific oyster extracts [21,22,23]. We reported that a DHMBA-rich diet drastically reduced hepatic steatosis and systemic obesity in high-fat–induced NASH model mice [24]. These results suggest a close relationship between oxidation and LDA in the liver, as previously reported. Activating Nrf2-dependent antioxidant activity in the liver might be a potential therapeutic target for the prevention of hepatic steatosis [21,25].

There are only a limited number of reports on the LDAI activity of food-derived metabolites, including β-carboline alkaloids [26,27,28,29]. Recently, researchers are paying attention to natural products, which may ameliorate lipid accumulation in hepatocytes. Previous studies reported that some extracts and compounds ameliorate the lipid accumulation in hepatocytes. Chinese olive extract ameliorated lipid accumulation through regulating lipid metabolism in the oleic acid-loaded mouse hepatocytes at 50–400 μg/mL in vitro, and in vivo in the hepatocytes of the oleic acid-treated mice [30]. Puerarin, an isoflavone compound isolated from an Asian herb, ameliorates hepatic steatosis in HepG2 cells at 100 μM through activating the peroxisome proliferator-activated receptor-alpha (PPAPα) and AMP-activated protein kinase (AMPK) signaling pathways [31]. Delphinidin, an anthocyanin derivative, also ameliorated hepatic triglyceride accumulation in human HepG2 at 180 μM [32].

**Figure 1:**
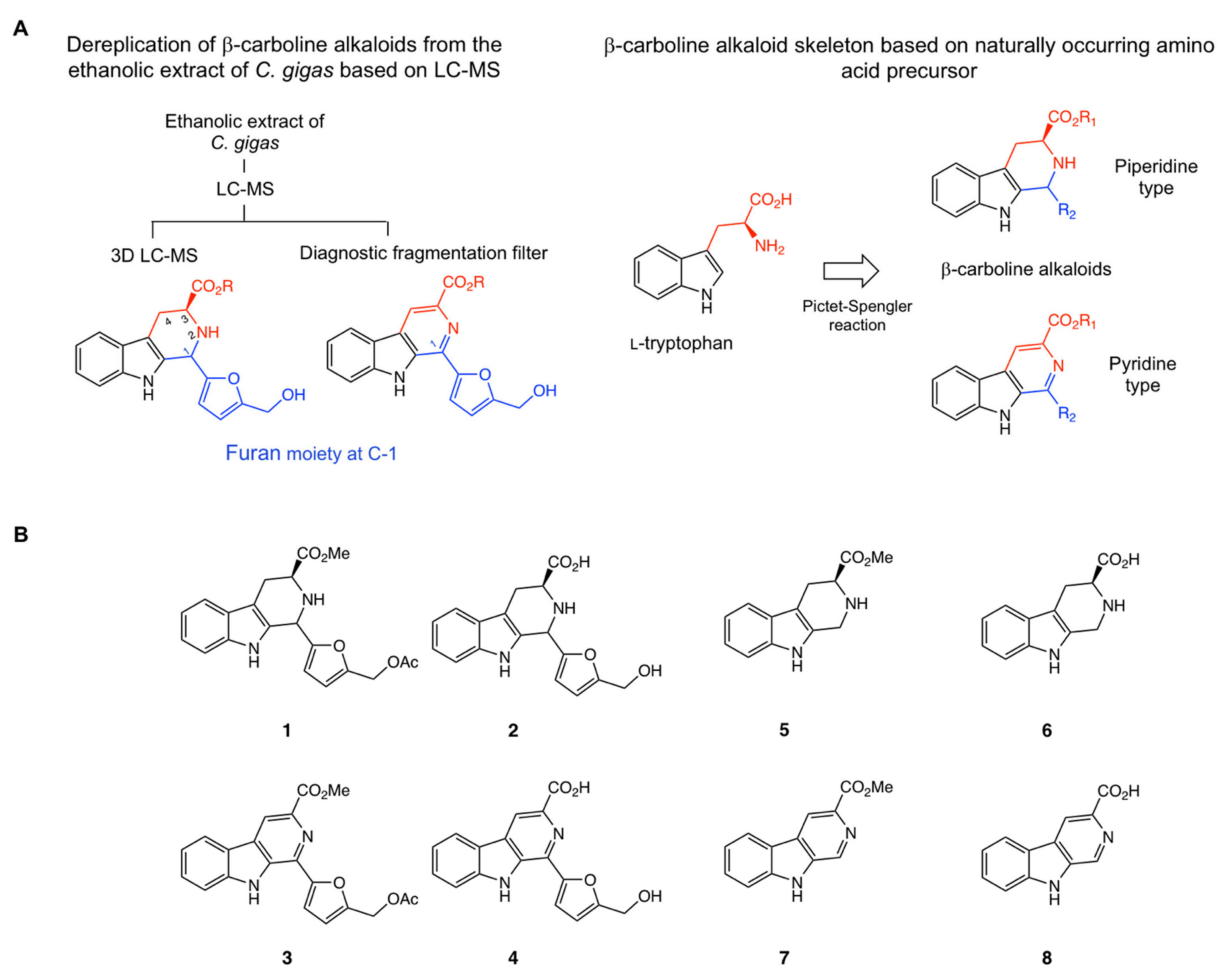
(**A**) Dereplication strategy of β-carboline alkaloids from *Crassostrea gigas* and design strategy for their structures; (**B**) Structures of designed β-carboline alkaloids **1**–**8**.

More interestingly, there are no reports on the association between LDAI activity and β-carbolines containing a pyran moiety substituted at C-1, such as flazin derivatives. Several β-carboline alkaloids containing a furan moiety with substitution at C-1 in minor constituents in ethanolic extracts of Pacific oysters were recently detected in this study. Based on the liquid chromatography/mass spectrometry (LC/MS) approach, flazin (**4**) containing a pyridine C-ring and its derivative compound containing a piperidine C-ring (**2**) as minor constituents were identified. The structure–LDAI relationship for these compounds has not yet been studied. Inspired by the two identified molecules, in this study, eight β-carboline alkaloid analogs containing a combination of a furan moiety at C-1 and a carboxy or methyl ester group at C-3 were designed and synthesized, using a one-pot Pictet–Spengler reaction (Figure 1). The precursor of these bioactive β-carboline alkaloids was a natural food-occurring aromatic amino acid, l-tryptophan [33]. The investigation of their LDAI activity and regulation of lipid metabolism in HepG2 cells that formed LDs upon supplementation with oleic acid were performed. The inhibition of accumulated triacylglycerol molecular species upon treatment with the identified bioactive food metabolites was quantified using an omics approach. The present study was aimed at determining the potential effects of various β-carboline alkaloids derived from food metabolites, including flazin, on LDA in hepatocytes.

## 2. Results and Discussion

### 2.1. Metabolite Fingerprinting of C. gigas and β-Carboline Alkaloid Identification

**Figure 2:**
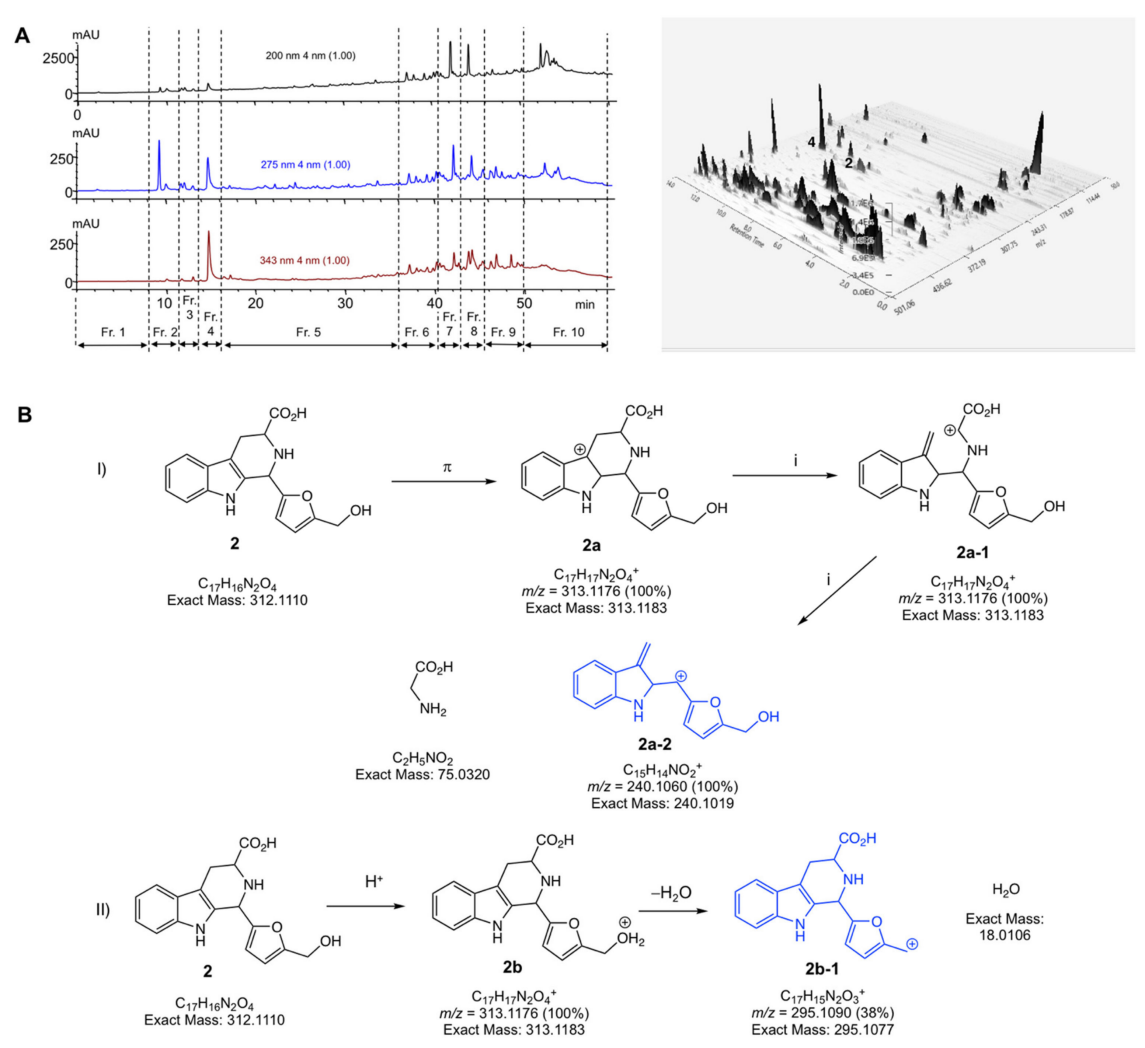
(**A**) HPLC and 3D LC/MS metabolite profiling of *Crassostrea gigas* ethanolic extract; (**B**) Proposed mass fragmentation pathway of compound **2** (Symbols: π referred to ionization on π bond, i: referred inductive cleavage, H^+^: protonation, −H_2_O: dehydration). MS/MS fragmentation showing, (I): Prominent product ion **2a** at *m/z* 240.1019 and mass neutral loss: 75.0320 (C_2_H_5_NO_2_); (II): Prominent product ion **2b-1** at *m/z* 295.1077 and neutral loss: 18.0106 (C_2_H_5_NO_2_).

*Crassostrea gigas* Thunberg is a nutrient-rich food and exhibits remarkable antioxidant activity, suggesting that it is a potential source of primary and secondary bioactive metabolites [21,22,23,24]. In this study, *C. gigas* samples were extracted with 70% EtOH to obtain an ethanolic extract. The semi-preparative HPLC of this extract using a water–MeOH gradient mixture gave ten fractions: Fr.1−Fr.10 (Figure 2A and Supplementary Materials, Figure S1). The LC/MS profiles of the ethanolic extract and all the collected fractions are presented as a 3D LC/MS plot with the retention time and MS values (Figure 2A and Supplementary Materials, Figures S1–S5).

The LC/MS measurement and analysis revealed the presence of the β-carboline alkaloid flazin (**4**), with a pyridine C-ring, and a flazin derivative, with a piperidine C-ring (**2**), in the oyster extract as minor constituents. It was possible to identify the two types of β-carbolines as chemical constituents of the ethanolic extract by comparing with the synthesized standards. As shown in the 3D plot in Figure 2A, flazin (**4**) and its derivative (**2**) were found in the extract based on the retention time and MS/MS spectra of the authentic synthesized standards; this is the first time that **2** has been identified in a *C. gigas* extract. Compound **2** showed mass loss found for heterocyclic-β-carboline indole derivatives and that can be explained by the elimination of NH_2_CH_2_COOH and H_2_O for the main ion products **2a** and **2b,** respectively. The mechanism of fragmentation for the main ion product of **2a-2** with an abundance of 100% was initiated by ionization of π bond at indole double bond and inductive cleavage (*i*). The ion product **2b** with an abundance of 38% was obtained by protonation of the hydroxy group on the furfuryl alcohol moiety, followed by elimination of H_2_O generating an ionic 2-methyl furan moiety at C-1 on **2b-1** (Figure 2B). The suggested mass fragmentation pathway of this β-carboline is well in agreement with similar compounds reported in literature [34,35,36,37,38]. The fragmentation behaviors of the predicted flazin derivative (**2**) with a piperidine ring having a furfuryl alcohol moiety at C-1 were proposed based on the observed MS^2^ fragment values (m/z); I, compound **2** exact mass: 312.1110 (C_17_H_16_N_2_O_4_); precursor ion (**2a**), MS^1^: 313.1176 (C_17_H_17_N_2_O_4_^+^); inducted ion (**2a-1**), MS^1^: 313.1176 (C_17_H_17_N_2_O_4_^+^); product ion (**2a-2**) MS^2^: 240.1060 (C_15_H_14_N_2_O_4_^+^); neutral loss: 75.0320 (C_2_H_5_NO_2_). II, precursor ion (**2b**), MS^1^: 313.1183 (C_17_H_17_N_2_O_4_^+^); product ion (**2b-1**) MS^2^: 295.1019 (C_15_H_14_N_2_O_4_^+^); neutral loss: 18.0320 (C_2_H_5_NO_2_) as shown in Figure 2B (Supplementary Materials, Figure S6). Thus, similar β-carboline alkaloids with piperidine C-ring with a furan moiety at C-1 in a food mixture can be detected by defining the specific MS/MS fragmentation criteria for the targeted compounds using the diagnostic fragmentation and neutral loss filter and the optimized instrument conditions. Diagnostic fragmentation filtering (DFF) is a straightforward and rapid strategy for detecting entire classes of compounds in a natural food product mixture; this is especially relevant for natural product compound dereplication and discovery [39].

### 2.2. Design and Synthesis of β-Carboline Alkaloid Metabolite Derivatives (Pyridine and Piperidine Types)

**Scheme 1:**
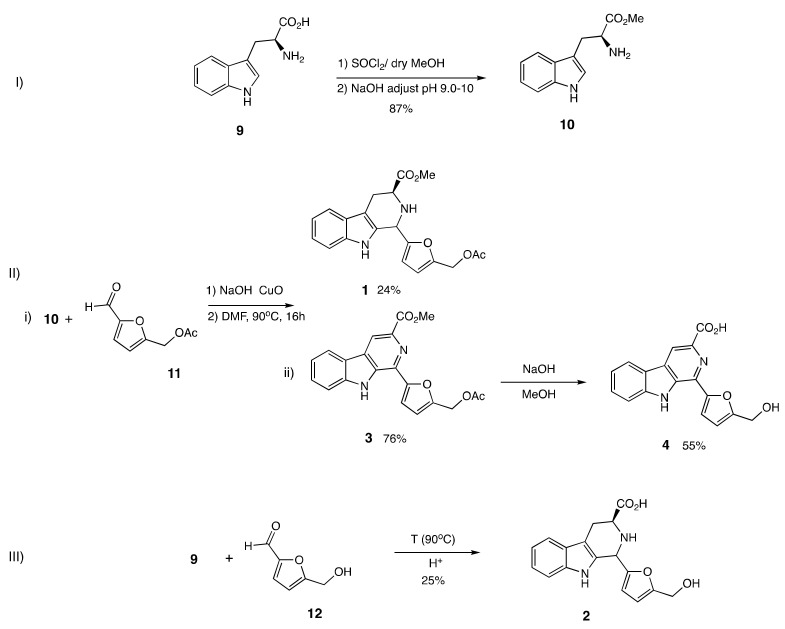
Synthesis of identified β-carboline alkaloids **2** and **4** with piperidine and pyridine ring, respectively. (**I**) Preparation of **10** by esterification; (**II**) (i) Preparation of **1** and **3** by one-pot Pictet–Spengler reaction; (ii) Preparation of **4** from **3;** (**III**) Preparation of **2** by acid conversion of aromatic amino acid tryptophan (**9**).

β-Carboline alkaloids form in foods through biosynthetic pathways and during cooking and storage. We have mimicked the production of β-carboline alkaloids during cooking and storage processes under acidic conditions using an aromatic amino acid as a precursor, and a naturally occurring food aldehyde. In this study, the two β-carboline alkaloids detected in ethanolic extracts of the functional food *C. gigas*, were chemically synthesized, along with other derivatives containing pyridine and a piperidine C-ring. Compounds **1**–**4** were substituted at C-1 with a furan moiety and at C-3 with a methyl ester or carboxy group. The compounds were prepared using a one-pot Pictet–Spengler reaction with CuO nanoparticles, as shown in Scheme 1 [33]. A commercially available food-occurring amino acid, l-tryptophan (**9**), was converted to compound **10**, methyl ester piperidine-type. An esterification reaction with appropriate dry MeOH and SOCl_2_ was used. Then, compound **10** was subjected to a one-pot Pictet–Spengler reaction with CuO nanoparticles in DMF at 90 °C for 16 h using aldehyde **11**, yielding two β-carboline alkaloids, **1** and **3**. Compound **3** was dissolved in methanol, and NaOH aqueous solution was added. The mixture was heated to 65 °C and stirred for 5 h. Then, citric acid was added to adjust the pH to 4−5. Then, the solution was extracted with ethyl acetate. The collected organic layer was purified to yield compound **4.** The aromatic amino acid tryptophan (**9**) and compound **12** were subjected to acid conversion at 90 °C in acetic acid to obtain compound **2** (Scheme 1). β-carboline alkaloids (**5**–**8)** without any substituted furan moiety at C-1 but containing a methyl ester or carboxy group at C-3 were also synthesized. Piperidine-types **5** and **6** were prepared by the acidic conversion of **10** and **9** with HCHO within 1 h. A one-pot Pictet–Spengler reaction with CuO nanoparticles was used to prepare pyridine types **7** and **8** from pyridine-type **5**. We then analyzed the importance of the effect of each feature on the synthetic alkaloids for LDAI activity

### 2.3. Cell Viability and LDAI Activity of β-Carboline Alkaloid Metabolite Derivatives

**Figure 3:**
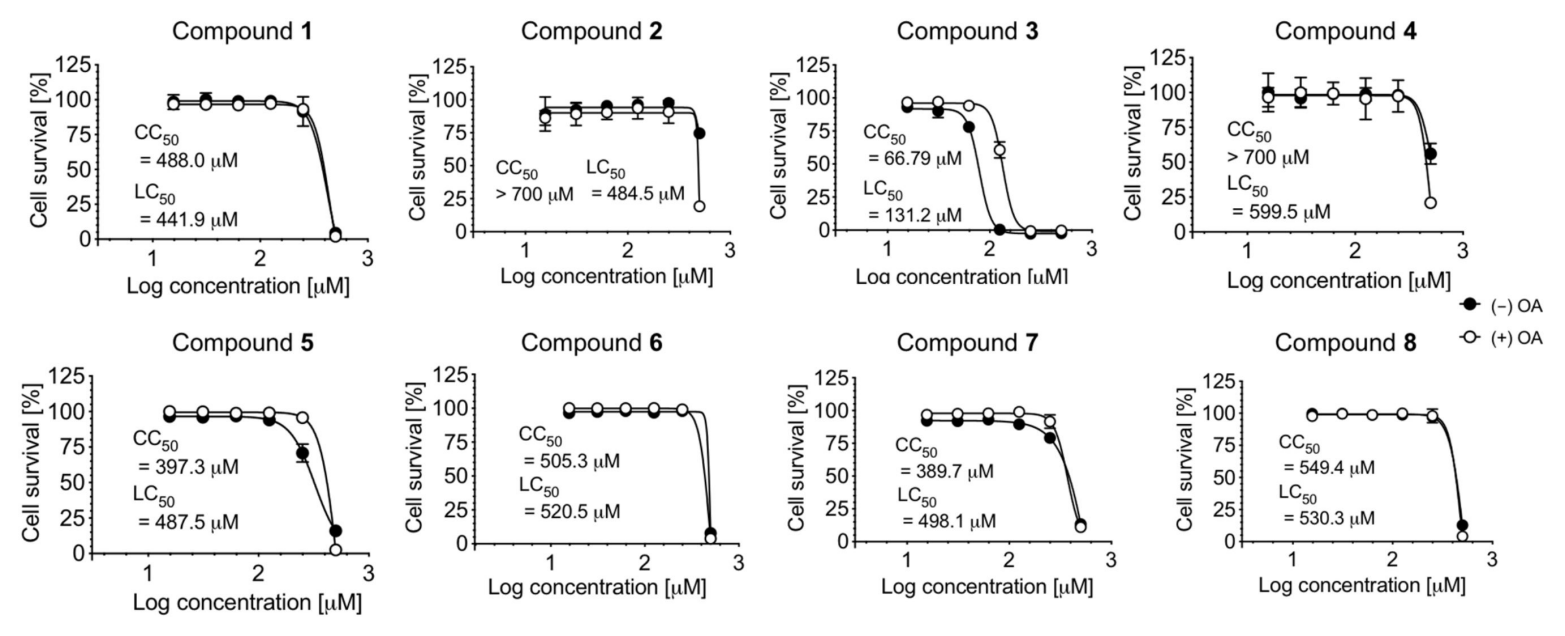
Cytotoxicity and lipotoxicity of **1**–**8** in HepG2 cells. The cytotoxicity (CC_50_) was expressed as the concentration at which 50% of cells died in DMEM condition without fatty acid (−OA). The lipocytotoxicity (LC_50_) was expressed as the concentration at which 50% of cells died, especially in DMEM condition loaded with fatty acid (+OA).

All the synthesized alkaloids were tested for cytotoxicity against the HepG2 cell line. The effects of the synthesized compounds on HepG2 cell viability are shown in Figure 3. The metabolites containing a carboxy group at C-3 and furfuryl alcohol moiety at C-1 showed an IC_50_ greater than 500 μM, whereas those with a methyl ester group were found to have low cytotoxicity values of less than 500 μM. Moreover, compound **3**, with pyridine C-ring compound containing a methyl ester group (-CO_2_Me) at C-3 and an acyl group on the furan moiety at C-1, significantly decreased cell viability by five-fold (66.9 μM) compared to the compound with piperidine C-ring (**1**, 488.0 μM) substituted to a methyl ester group (-CO_2_Me) at C-3 and acyl group on the furan moiety at C-1. This suggested that the combination of a methyl ester, pyridine C-ring, and acyl group on the furan moiety is responsible for the pronounced observed cytotoxicity of compound **3** (Figure 3).

**Figure 4:**
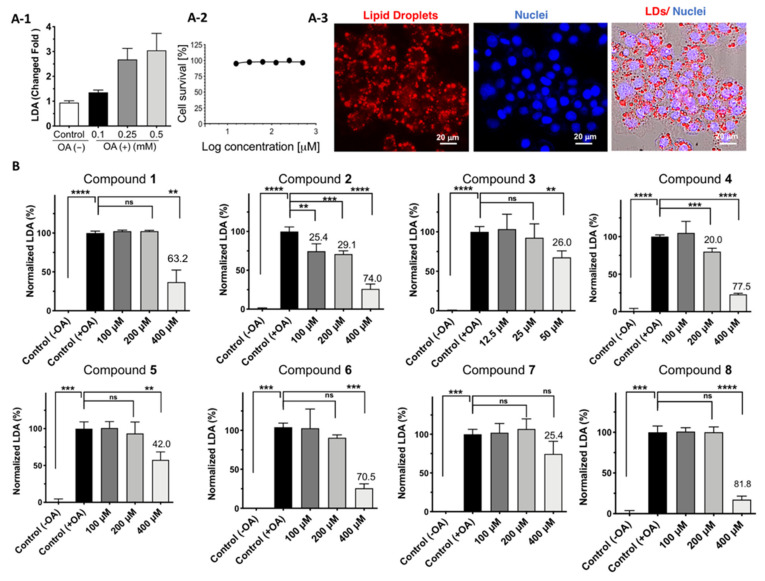
(**A**) Lipid droplet accumulation (LDA). A-1: LDA induced by 0.1, 0.25, and 0.5 mM oleic acid in HepG2 cells; A-2: Cell survival with oleic acid (0.25 mM); A-3: Cell survival with oleic acid (0.25 mM); (**B**) LDA inhibition (LDAI) activity of **1**–**8** in HepG2 cells. Graph showing the mean values of the LDAI (four replications). **** *p* < 0.0001, *** *p* < 0.001, ** *p* < 0.01 when compared with the untreated control (+OA) group. ns: not significant.

LDs contain TAGs as their hydrophobic cores and are enclosed by a phospholipid monolayer, mainly phosphatidylcholine (PC), a hydrophilic shell. LDs can grow on the bilayer membrane of the endoplasmic reticulum [40]. The intracellular accumulation of LDs is related to various metabolic diseases in humans. Reduction in LDA could prevent metabolism-related disorders. Natural and synthetic LDA inhibitors are emerging as potential bioactive candidates against obesity-associated NAFLD. In the present study, all the synthesized β-carboline alkaloid compounds (**1**–**8**) were tested for their LDAI potency in HepG2 cells in DMEM supplemented with oleic acid. LDs were first induced by growing HepG2 cells after incubation for 24 h in DMEM supplemented with 0.1, 0.25, and 0.5 mM oleic acid (Figure 4A1–3) to determine the appropriate concentration for the study. LDs were induced significantly, by 2.6 and 2.8-fold, with 0.25 and 0.5 mM oleic acid, respectively. Thus, 0.25 mM of oleic acid was chosen to induce LDs in HepG2 cells.

Moreover, the HepG2 cells were tested for cell viability after treatment with 0.25 mM oleic acid and no cytotoxicity was observed after 24 h (Figure 4A-1). All the chemically synthesized alkaloid compounds were then tested for LDAI potency at their non-toxic concentration ranges in HepG2 cells. HepG2 cells supplemented with oleic acid were exposed to all synthesized compounds at 100, 200, and 400 μM and incubated for 24 h, except for compound **3** at 12.5, 25, and 50 μM. The compounds with the carboxy group (-COOH) at C-3 and furfuryl alcohol moiety at C-1 (IC_50_ > 400 μM) showed more significant inhibition of LDA compared to their corresponding methyl ester derivatives (-COOMe) (Figure 4B). Compounds **2**, and **4** were found to selectively inhibit LDA in a concentration-dependent manner in HepG2 cells at non-toxic concentrations (**2**, LDAI: 25.5%; 100 μM and 29.1%; 200 μM and **4**, LDAI: ns; 100 μM and 20.0%; 200 μM). In contrast to compound **3**, which showed no dose-response on LDA inhibition but a trend toward inhibition at the low concentration tested, compound **3**, with a pyridine C-ring, connected to the acetoxy furan group at C-1 and methyl ester group (-COOMe) at C-3, showed pronounced cytotoxicity and exhibited an LDAI inhibition (26.0%; 50 μM), but no significant LDAI at the selected concentration range (12.5 and 50 μM). Based on this observation, we deduced a consistent relationship between structure and LDAI activity. Piperidine and pyridine C-ring with a C-3 carboxyl group (-COOH) and connected to furfuryl alcohol moiety at C-1 showed enhanced inhibition with less cytotoxicity (Figure 3 and Figure 4B). β-carboline alkaloid flazin (**4**), and its derivatives containing a C-3 carboxyl group and a C-1 furan moiety, have a natural origin and are also derived from food processes [41,42,43,44,45,46,47,48]. Because compounds **2** and **4** have similar features at C-1 and C-3, they were selected for detailed investigation to understand the importance of their C-ring: fully saturated tetrahydro-b-carboline at 1, 2, 3, in **2** (piperidine C-ring) and unsaturated β-carboline in **4** (pyridine C-ring) on LDAI activity.

Furthermore, to visualize the inhibition of the accumulated neutral LDs, we performed real-time LD formation monitoring of control −OA, +OA, and LDAI experiments on flazin in HepG2 cells loaded with oleic acid. All cell morphological changes and LD events were observed for 20 h. LD formation was observed at 6 h, and significant accumulation of LDs was achieved 12, 18, and 20 h after incubation, as shown by the live imaging of LDA and Oil Red O staining (Figure 4A and Supplementary Materials, Figure S12). LDA was observed in the control cells, which emitted green fluorescence exclusively in the AO/EB staining experiment, indicating live cells even after co-culture with oleic acid for 20 h (Supplementary Materials, Figure S12). This experiment showed the formation of LDs over 24 h; the cells were still alive at the end of the experiment during LDA. Moreover, LDAI by flazin (**4**) in HepG2 cells grown on DMEM with oleic acid was monitored in real-time, and a movie was created, as shown in Video S1 SI2-Part 2 (Supplementary Materials). LDA was inhibited at 6 h but was significant at 12 and 20 h after treatment with flazin (**4**), as shown in the capture of the live imaging. HepG2 cells in the control supplemented with OA continued to exhibit division, proliferation, and LDA. In the treated sample, the accumulated neutral LDs significantly reduced in number within 20 h. During real-time imaging, a significant attenuation of LDA was observed between 6 and 20 h, with a clear change in the morphology and size of LDs compared with those in the control at the corresponding time points (Supplementary Materials, Figures S13–S15).

### 2.4. Quantification of Accumulated Triacylglycerol Species Inhibition

**Figure 5:**
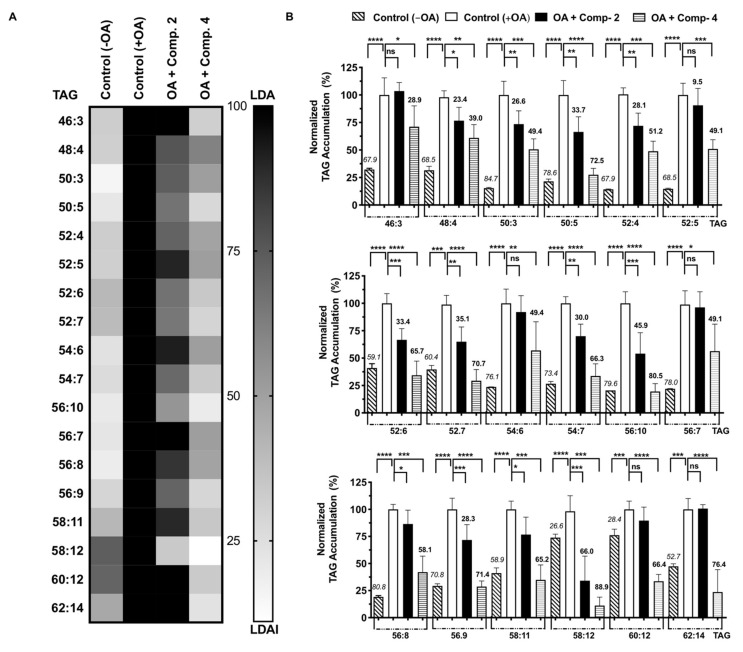
(**A**) Heat map of accumulated and inhibited TAG species in cells; (**B**) Quantification of fluctuation on accumulated TAG species. The italicized and bold values at the top of the columns represent the accumulation and inhibition of each species compared to its negative (−OA) and positive (+OA) and control, respectively. Graph showing the mean values of the LDA and LDAI (six replications) **** *p* < 0.0001, *** *p* < 0.001, ** *p* < 0.01, * *p* < 0.05 when compared with the untreated control (+OA) group. ns: not significant.

NAFLD is the most common liver disease associated with obesity. It is characterized by excess LDA in hepatocytes. LDs are spherical organelles that mainly store intracellular neutral lipids, such as triacylglycerols and cholesterol esters. OA-treated HepG2 cells showed a significant increase in LDA, and LDAI was observed for compounds **2** and **4**, as shown in Figure 4. The bioactive compounds **2** and **4** inhibited LDA in a concentration- dependent manner in HepG2 cells loaded with oleic acid. We analyzed the effect of food-derived β-carboline alkaloids **2** and **4** on triacylglycerol species accumulation in hepatocytes using an LC-MS orbitrap. Seventy-five triacylglycerol molecular species were detected using *in-house* system established lipidomics. More than thirty species were accumulated. Eighteen accumulated species were inhibited by compounds **2** and **4** (Figure 5A). Compounds **2** and **4** inhibited twenty-five and twenty triacylglycerol molecular species accumulated in cells after 24 h of treatment at 200 μM, respectively. Most of the accumulated triacylglycerol molecular species were inhibited by compound **2** in the range of 25% to 49%, except for TAGs 58:12; 46:3; 54:6; and 56:7. Six accumulated triacylglycerol molecular species (TAGs: 46:3; 48:8; 50:3; 52:5; 54:6; and 56:7) were inhibited by compound **4**. In the range of 50–74%, the accumulated triacylglycerol molecular species (TAG 58:12) was particularly inhibited by compound **2**.

Ten accumulated triacylglycerol molecular species were inhibited by compound **4** (TAGs 50:5; 52:4; 52:6; 52:7; 54:7; 56:10; 56:9; 58:11; 50:12; and 62:4). In the range between 50% and 74%, inhibition was significantly observed, especially by compound **4**, for two accumulated triacylglycerol molecular species (TAGs 56:10 and 58:12). Among all the accumulated triacylglycerol molecular species, total inhibition was observed only for TAGs 52:6; 52:7; 56:10; 56:9; and 62:4 during flazin (**4**) treatment (Figure 5B). Compounds **2** and **4** ameliorated LDA through inhibiting the accumulation of triacylglycerol molecular species in human HepG2.

### 2.5. Gene Expression

The food-derived compounds **2** and **4** inhibited LDA in HepG2 cells loaded with OA at 200 μM. In the fluorescence imaging experiments, the red (LDs: lipid droplets) were stained with Oil Red O, and the nuclei were stained blue with Hoechst 33,342 dye. The fluorescence image showed that the LDs were significantly inhibited by compounds **2** and **4** (Figure 5A). Gene expression analysis to investigate the effects of compounds **2** and **4** on lipolysis and lipogenesis were performed. Either compound **2** or **4** were supplemented under LDA conditions in HepG2 cells and analyzed lipolysis- and lipogenesis-related gene expression. First, adipose triglyceride lipase *(ATGL)*, which plays a critical role in the hydrolysis of TGs, was upregulated upon supplementation of compounds **2** and **4** under conditions of LD formation. Therefore, compounds **2** and **4** could reduce LDA by downregulating *ATGL*. On the other hand, *DGAT1*, an enzyme involved in TG synthesis, showed no change among the groups.

**Figure 6:**
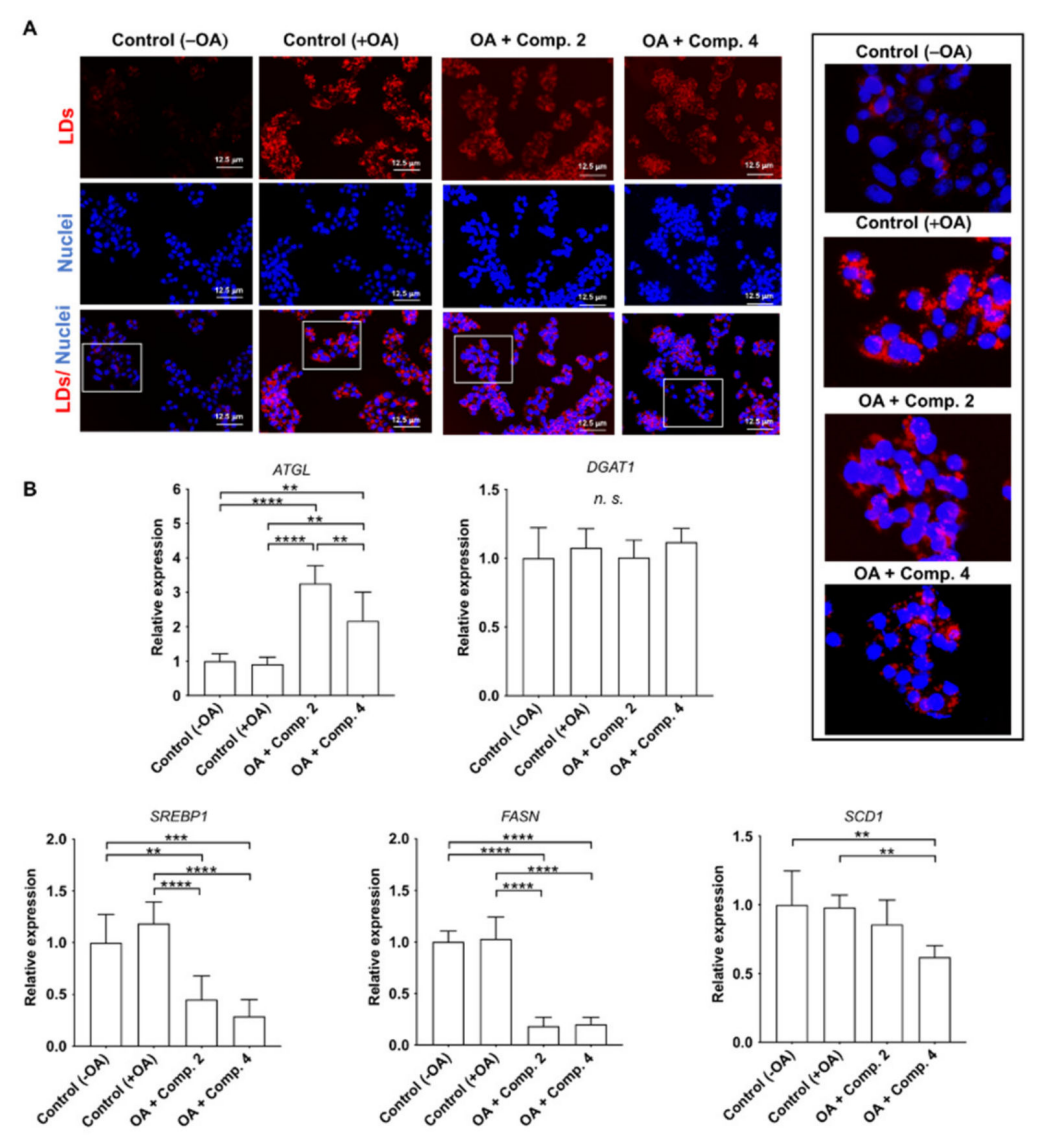
(**A**) LDAI activity of compounds **2** and **4** in HepG2 cells: fluorescence images showing LDs (red) and nuclei (blue); (**B**) Gene expression. All data were normalized to the expression of glyceraldehyde-3-phosphate dehydrogenase (*GAPDH*) and were represented as the relative expression based on that for the control group. *ATGL*: adipose triglyceride lipase; *DGAT1*: diacylglycerol O-acyltransferase 1; *SREBP1*: sterol regulatory element-binding protein 1; *FASN*: fatty acid synthase; *SCD1*: stearoyl-CoA desaturase 1. ** *p* < 0.01, *** *p* < 0.001, **** *p* < 0.0001 (*n* = 6 in each group).

**Figure 7:**
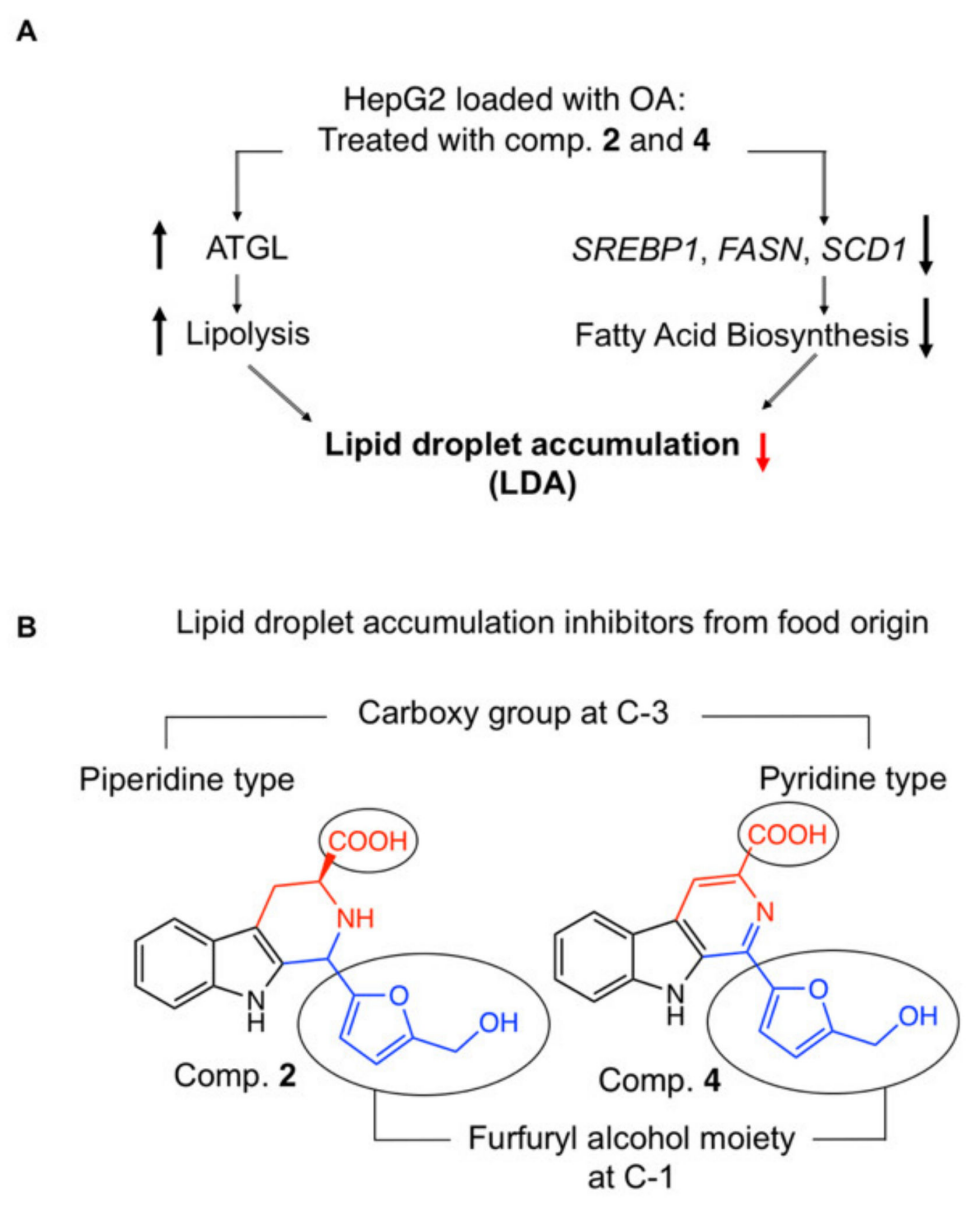
(**A**) Proposed LDAI activity pathway of compounds **2** and **4** in HepG2 cells; (**B**) Chemical structure features of food-derived LDA inhibitors **2** and **4** important for LDAI activity.

Next, the expression of sterol regulatory element-binding protein 1 *(SREBP1)*, fatty acid synthase *(FASN)*, and stearoyl-CoA desaturase 1 *(SCD1)*, which are related to de novo lipogenesis including fatty acid biosynthesis, were investigated. Upon compound **4** supplementation, the expression of all these genes was strongly downregulated. Compound **2** did not induce a significant reduction in *SCD1* expression, but significantly downregulated *SREBP1* and *FASN* (Figure 6B). Therefore, compounds **2** and **4** are also associated with the suppression of fatty acid biosynthesis. These results indicate that compounds **2** and **4** may inhibit LD formation by suppressing fatty acid synthesis and upregulating lipolysis (Figure 7A). A clear structure–activity relationship was deduced for all the tested compounds (**1**–**8**) with regard to LDAI. The combination of the carboxy group at C-3 and the furan methyl hydroxy group at C-1 on both the piperidine and pyridine rings of compounds **2** and **4** is the main chemical feature responsible for the observed LDAI activity (Figure 7B). A systematic investigation of the bioavailability of related metabolites with different skeletons in various medicinal plants, foods and after food processing is needed to identify more potent metabolites and to fully understand their structure–activity relationship.

## 3. Materials and Methods

### 3.1. Chemicals and Materials

General Experimental Procedures: NMR spectra were recorded using a JEOL ECX400 Delta spectrometer with TMS as an internal standard, and chemical shifts were expressed as *δ* values. An LTQ Orbitrap XL (Thermo Fisher Scientific Inc., San Jose, CA, USA) mass spectrometer was used for high-resolution HR-ESI-MS measurements. Low-resolution electrospray ionization mass spectrometry (LR-ESI-MS) spectra were recorded using an LXQ spectrometer (Thermo Fisher Scientific Inc., San Jose, CA, USA). Analytical thin-layer chromatography (TLC) was performed on pre-coated silica gel 60F_254_ and RP-18F_254_ plates (0.25 or 0.50 mm, Merck KGaA, Darmstadt, Germany). Preparative TLC was performed on pre-coated silica gel 70 PF_254_ plates (0.75 mm thickness; Wako Pure Chemical Industries, Ltd., Osaka, Japan). Semi-preparative HPLC separations were performed on a Shimadzu quaternary LC VL instrument with a UG 120 A column (250 × 10 mm i.d.; 5 μm). Methanol was purchased from Wako. High-glucose Dulbecco’s Modified Eagle Medium (DMEM), Dulbecco’s phosphate-buffered saline (DPBS), trypsin EDTA, fetal bovine serum (FBS), and penicillin–streptomycin (100 U/mL) were purchased from Gibco (Life Technologies, Carlsbad, CA, USA). Other materials used for cell culture were purchased from Corning (NY, USA). TLC silica gel 60G F_254_ glass plates (20 × 20 cm) were obtained from Merck (Tokyo, Japan), and spots were visualized by spraying with 5% ethanolic H_2_SO_4_. ^1^H, ^13^C NMR, DEPT, and 2D NMR spectra were acquired using a 400 MHz JNM-ECX400P spectrometer (JEOL, Tokyo, Japan). The spectra were processed using JOEL software, and the chemical shifts (*δ*) values were expressed in ppm. The EquiSPLASH LIPIDOMIX^®^ Quantitative Mass Spec Internal Standard (Avanti Polar Lipids, Alabaster, AL, USA) was used as the internal standard for lipidomics of the neutral lipids. The mobile phases for LC/MS were Ammonium Acetate (Wako Pure Chemical, Osaka, Japan) and LC grade methanol (Kanto Chemical, Tokyo, Japan). Oleic acid (OA) was purchased from Cayman Chemical (Ann Arbor, MI, USA), and Oil Red O and 2-Propanol were purchased from Wako Pure Chemical (Osaka, Japan). The absorbance was measured at 490 nm using ARVO-MX (Perkin Elmer, Waltham, MA, USA).

### 3.2. Metabolite Fingerprint of C. gigas and β-Carboline Alkaloid Dereplication

#### 3.2.1. HPLC and LC/MS Profiling

Pacific oyster (*C. gigas*) samples were extracted with 70% EtOH in three cycles at room temperature. The extracts were combined and concentrated under reduced pressure to yield an ethanolic extract. The ethanolic extract was passed through silica gel under HPLC using a water–MeOH gradient mixture; thus, ten fractions were obtained (Fr.1, 9 min; Fr.2, 11 min; Fr.3, 14 min; Fr.4, 16 min; Fr.5, 36 min; Fr.6, 41 min; Fr.7, 43 min; Fr.8, 45 min; Fr.9, 51 min; Fr.10, 60 min) (Figure 2). The ethanolic extract and all ten fraction samples were analyzed using a Shimazu LC system (Shimadzu Corporation, Kyoto, Japan) coupled to an LTQ Orbitrap XL (Thermo Fisher Scientific Inc., San Jose, CA, USA) mass spectrometer. Samples were separated using an Atlantis T3 C18 column (2.1 × 150 mm, 3 μm, Waters, Milford, MA, USA) at a flow rate of 200 μL/min, and the column and sample tray were maintained at 40 °C and 4 °C, respectively. An injection volume of 10 μL and gradient elution program with 10 mM ammonium acetate solution, isopropanol, and methanol were used for chromatographic separation. A heated electrospray ionization (HESI) source with positive ionization modes was used for both HREIMS and HREIMS/MS analyses. The MS data were acquired in ESI-positive mode. A Fourier-transform (FT) full scan range of *m*/*z* 50–500 was set to acquire MS^1^ spectra for high-resolution masses. MS/MS spectra were obtained by data-dependent acquisition using collision-induced dissociation (CID) in the ion-trap mode for low-resolution masses. The obtained raw data were processed using Xcalibur 2.2 (Thermo Fisher Scientific Inc., San Jose, CA, USA) with a mass tolerance of 5.0 ppm. HREIMS feature detection was performed using MZmine 2 (http://mzmine.github.io). The RT and MS values of the metabolites identified in the Pacific oysters were compared with synthesized standards **2** and **4**. The Vender software Xcalibur was used for analysis of results and metabolite identification.

#### 3.2.2. Synthesis Procedure

The natural food-occurring aromatic amino acid l-tryptophan (**9**) was used as a precursor of the designed β-carboline alkaloids. Synthesis of compound **10**: A stirred solution of tryptophan (**9**; 500 mg, 2.45 mmol) in 10 mL of dry MeOH, SOCl_2_ (0.8754 mL, 30 mmol) was added dropwise within 10 min under ice cooling. The mixture was stirred for 3 h, the solvent was removed under reduced pressure, and H_2_O (25 mL) was added. Then, the solution was extracted with AcOEt after being adjusted to pH 9–10 with aq. NaOH solution. The organic layer was washed with brine, filtered, and evaporated to yield compound **10** (Scheme 1, ^1^H, and ^13^C, see Supplementary Materials: Figure S7). A one-pot Pictet–Spengler reaction was used to synthesize the β-carboline alkaloid derivatives (**1**–**4**) with a piperidine ring and pyridine C-ring connected at C-1 with a furan moiety [34]. (Scheme 1, ^1^H, and ^13^C; see Supplementary Materials: Materials and Methods, Figures S7–S11 and Table S1). 5-Hydroxymethyl-2-furaldehyde (**12**) (296.7 mg; 1.57eq; 2.36 mmol) was added to l-tryptophan (**9**; 306 mg; 1.5 mmol) in acetic acid (30 mL). The entire solution was observed. The mixture was refluxed (90 °C); after 3 h, LR-MS showed conversion to compound **2**, and the reaction was completed. The mixture was cooled to room temperature and concentrated to dryness. Thus, a yellow solid was obtained. This solid was purified in a silica gel chromatography column to compound **2**. The aromatic amino acid, tryptophan (**9**) and compound **12** were subjected to acid conversion at 90 °C in acetic acid to obtain compound **2**. Compound **4** was dissolved in methanol, and NaOH aqueous solution was added. The mixture was heated to 65 °C and stirred for 5 h. Afterward, citric acid was added to adjust the pH to 4−5. The solution was extracted with ethyl acetate. The collected organic layer was purified to yield compound **2**. We also prepared similar compounds without any substituent at the C-1 position in the C-ring using a one-pot Pictet–Spengler reaction (**6**–**8**). We investigated the structure–LDAI relationship for all the series of synthetic β-carboline alkaloids (**1**–**8**): Methyl 1,2,3,4-Tetrahydro-1-(5-[(acetyloxy)methyl]-2-furanyl)-9H-pyrido [3,4-b]indole-3-carboxylic acid (**1**); 1,2,3,4-tetrahydro-1-(5-(hydroxymethyl)-2-furanyl)-9H-pyrido [3,4-b]indole-3-carboxylic acid (**2**); flazin methyl ester acetate (**3**); flazin (**4**); methyl 1,2,3,4-tetrahydro-β-carboline-3-carboxylate (**5**); 1,2,3,4-Tetrahydro-β-carboline-3-carboxylic acid (**6**); methyl β-carboline-3-carboxylate (**7**); and β-Carboline-3-carboxylic acid (**8**). β-carboline alkaloids compound (**1**–**8**) (Materials and Methods, in Supplementary Materials).

### 3.3. Cell Culture and Cell Viability Assay

HepG2 cells were purchased from the American Type Culture Collection (ATCC) (Manassas, VA, USA). High-glucose DMEM containing 10% heat-inactivated FBS and 1% penicillin–streptomycin was maintained under a humidified atmosphere at 37 °C with 5% CO_2_, as indicated in the Supplementary Materials. HepG2 cells (1.5 × 10^4^/well) in DMEM supplemented with 10% FBS were seeded into a 96-well plate. A cytotoxicity assay was performed according to the manufacturer’s protocol using CCK-8 (Dojindo Molecular Technologies), as indicated in the Supplementary Materials (Materials and Methods).

### 3.4. LDAI Assay, LD Fluorescence Staining Assay, and Real-Time LDAI Monitoring

LDAI assay: LDAI activity was determined using an Oil Red O assay with 24-well plates (*n* = 4 per treatment). Staining of LDs in cultured hepatocytes was performed according to the manufacturer’s instructions, based on the Oil Red O staining assay. Briefly, HepG2 cells (1.5 × 10^4^/well) were supplemented with 10% FBS, cultured, seeded in 35-mm dishes, and treated with the tested samples after 24 h. Oil Red O, a fat-soluble dye, is widely used for staining neutral lipids in LDs, as described in the Supplementary Materials. Next, quantification of LD inhibition was assessed for all test compounds **1** to **8** by comparing them to the untreated control group (+OA) and normalizing the LDA absorbance values (%) (Materials and Methods, in the Supplementary Materials).

LD fluorescence staining assay: The staining was performed with modifications based on our previous report [39]. HepG2 cells were seeded in 35 mm glass bottom dishes at 4.0 × 10^5^/plate. After 24 h of incubation, OA and all samples were processed for 24 h. Oil Red O staining was performed, then the Hoechst reagent solution mixed with PBS was added and incubated in the dark for 25 min. Images were then captured using a BZ-9000 fluorescence microscope (Keyence Co., Ltd., Osaka, Japan).

Real-time LDAI monitoring: Morphological changes in HepG2 cells and LDs induced by treatment with **4** were monitored using a Nikon, Hamamatsu Photonics/Ti-E, CCD Camera for 20 h. The cell viability of the control cells was examined by staining with acridine orange (AO)/ethidium bromide (EB) dyes at the end of the experiment (20 h) (Supplementary Materials, Figure S12).

### 3.5. Analysis of Accumulation on Triacylglycerols Species by LC-MS/MS

Tested compounds (200 μM) were added to cultured HepG2 cells loaded with oleic acid and incubated at 37 °C for 24 h, as previously described. Centrifuged samples were analyzed using an LC/MS orbitrap. Chromatographic separation was performed using a Shimadzu Prominence UHPLC system with a binary solvent delivery system and a standard auto-sampler. An Atlantis^®^ T3 column (2.1 × 150 mm, 3 μm, Waters) was used for separation, with a flow rate of 200 μL/min. The mobile phase consisted of A: 10 mM ammonium acetate solution; B: isopropanol; and C: methanol. Positive mode: 0–1 min, 6% B and 90% C; 1–10 min, 83% B and 15% C; 10–19 min, 83% B and 15% C; 19–19.5 min, 6% B and 90% C; 19.5–22 min, 6% B and 90% C. The injection volume was 10 μL. The column temperature was maintained at 40 °C. The LC/MS parameters were as previously described [40].

### 3.6. Lipid Metabolism–Related Gene Expression

To better understand how the supplementation of compounds **2** and **4** during LDA in HepG2 cells affects lipolysis and lipogenesis, we performed gene expression analysis using real-time PCR under the following conditions. Briefly, HepG2 cells (2.0 × 10^5^/well) were seeded in 24-well plates (*n* = 6 per treatment). After 24 h, the cells were treated with PBS, 0.25 mM OA only, 0.25 mM OA plus 200 and 400 μM compound **2**, or 0.25 mM OA plus 200 and 400 μM compound **4**. After another 24 h, RNA extraction and complementary DNA synthesis were performed, according to the manufacturer’s instructions and a previous study [49]. The sequences of the primers used are shown in the Supplementary Materials, Table S1: adipose triglyceride lipase (*ATGL*), diacylglycerol O-acyltransferase 1 (*DGAT1*), sterol regulatory element-binding protein 1 (*SREBP1*), fatty acid synthase (*FASN*), stearoyl-CoA desaturase 1 (*SCD1*), and glyceraldehyde-3-phosphate dehydrogenase (*GAPDH*). PCR was performed using a CFX 96 Real-Time PCR Detection System (Bio-Rad Laboratories Inc., Hercules, CA, USA) under the following conditions: 95 °C for 30 s, followed by 40 cycles at 95 °C for 5 s, 60 °C for 5 s, and 72 °C for 10 s. All gene expression levels were analyzed using the ΔΔCt method and normalized to *GAPDH*. Data were represented as relative expression based on that of the control group.

### 3.7. Statistical Analysis

For gene expression analysis, mean values were compared using one-way analysis of variance (ANOVA) followed by Tukey’s multiple comparison test. Statistical analyses were performed using GraphPad Prism 8.0e (GraphPad Software Inc., La Jolla, CA, USA). Statistical significance was set at *p* < 0.05.

## 4. Conclusions

In conclusion, a series of synthetic β-carboline alkaloids, inspired by flazin and its derivative, identified in Pacific oyster (*C. gigas*) extract, were investigated for their ability to inhibit LDA, using HepG2 cells loaded with fatty acids. Among them, compounds **2** and **4**, β-carboline alkaloids with a carboxy group at C-3 and furfuryl alcohol moiety at C-1, showed no cytotoxicity. Compounds **2** and **4** showed significant LDAI activity. Moreover, these compounds induced a drastic change in cell morphology. The bioactive compounds **2** and **4** inhibited the accumulation of triacylglycerol species in HepG2 cells under oleic acid-loaded conditions. Total inhibition was exhibited by flazin (**4**) for TAGs 52:6; 52:7; 56:10; 56:9; and 62:4. Compounds **2** and **4** ameliorated LDA through inhibiting the accumulation of triacylglycerol molecular species in human HepG2. A genetic study suggested that compounds **2** and **4** exerted LDAI activity by upregulating lipolysis and suppressing fatty acid biosynthesis. Compound **2** was identified for the first time in *C. gigas* extract by the LC-MS/MS approach and ameliorated the lipid accumulation in human hepatocytes. The identified β-carboline alkaloids with LDAI activity may be useful for the prevention and management of NAFLD/NASH.

## Data Availability

Data is contained within the article and Supplementary Materials.

## Supplementary Materials

The following supporting information can be downloaded at: https://www.mdpi.com/article/10.3390/ph15050578/s1, Materials and Methods, HPLC chromatograms, and 3 D plot of LC-MS metabolite profiling of Pacific oyster fractions (Fr1–Fr10) in negative and positive modes (Figures S1–S5); MS/MS spectra of compound 2: Proposed mass fragmentation of compound 2. (Figure S6); spectroscopic data for 1–4 and 10 (Figures S7–S11), and HREIMS data of 1–8 in positive mode (Table S1). The captures of a live imaging experiment showing the LDAI effect of flazin (4) on hepatocytes (HepG2 cells) at interval of 6h and lipid droplet staining by Oil red. Cell viability and morphological stain by AO/EB (Figure S12). The captures of a live imaging experiment showing the LDs formation and LDAI effect of flazin (**4**) on HepG2 cells (Figures S13–S15: −OA, +OA, and treated **4**) (PDF). Video S1: Real-time movie showing the LDs formation and LDAI effect of flazin (**4**) on hepatocytes (HepG2 cells) with one frame per 15 min for 20 h (AVI). List of primers (Table S2).

## Abbreviations

LDA: lipid droplet accumulation; LDAI, lipid droplet accumulation inhibition; NAFLD, nonalcoholic fatty liver disease; NASH, nonalcoholic steatohepatitis; oxLD, oxidized lipid droplet; OA, oleic acid; LA, linoleic acid; EB, ethidium bromide; AO, acridine orange; LD, lipid droplet; TAGs, triacylglycerols; PC, phosphatidylcholine; AAPH, 2,2′-azobis(2-amidinopropane) dihydrochloride; DHMBA, 3,5-dihydroxy-4-methoxybenzyl alcohol; LC/MS, liquid chromatography/mass spectrometry; NMR, nuclear magnetic resonance; HPLC, high-performance liquid chromatography; ATGL, adipose triglyceride lipase; DGAT1, diacylglycerol O-acyltransferase 1; SREBP1, sterol regulatory element-binding protein 1; FASN, fatty acid synthase; SCD1, stearoyl-CoA desaturase 1; GAPDH, glyceraldehyde-3-phosphate dehydrogenase.
