# Supplementary material for: Food-Derived β-Carboline Alkaloids Ameliorate Lipid Droplet Accumulation in Human Hepatocytes
Source: Pharmaceuticals (Basel). 2022 May 5;15(5):578. doi: 10.3390/ph15050578 (PMC9147645; doi:10.3390/ph15050578)
Supplement: Supplementary file 1 [file pharmaceuticals-15-00578-s001.zip › Supporting Information Part 1 (SI Part 1).pdf]

Supporting Information Part 1 (SI Part 1)

Food-derived β-carboline alkaloids ameliorate lipid droplet accumulation in human hepatocytes

Dya Fita Dibwe,[1] Saki Oba,[1] Nire Takeishi,[1] Toshihiro Sakurai,[1] Takayuki Tsukui,[2] Hitoshi Chiba H,[2] Shu-Ping Hui *,[1]

[1]Faclty of Health Sciences, Hokkaido University, Kita-12, Nishi-5, Kita-Ku, Sapporo 060-0812, Japan

[2]Department of Nutrition, Sapporo University of Health Sciences, Nakanuma Nishi-4-3-1-15, Higashi-Ku, Sapporo 007-0894, Japan

Table of contents:

1. Supporting Information Part 1-1

1. Material and Methods P5

1.1 Synthesis procedure P5

1.2 Cytotoxicity P5

1.3 Lipid droplet accumulation inhibition P5

1.4 Oil Red O Staining. P5

1.5 Fluorescent Imaging Analysis P5

Figure S1: Fractionation of ethanolic extract of Pacific oyster (*Crassostrea gigas*) P6

Figure S2: 3D plot of LC-MS metabolite profiling of Pacific oyster fractions (Fr1,Fr2, Fr4-Fr7) fractions in positive mode P7

Figure S3: 3D plot of LC-MS metabolite profiling of fractions (Fr8-Fr10; Merge: Fr1-10) of Pacific oyster (*Crassostrea gigas*) in positive mode P8

Figure S4: 3D plot of LC-MS metabolite profiling of Pacific oyster fractions (Fr1,Fr2, Fr4-Fr7) fractions in negative mode P9

Figure S5: 3D plot of LC-MS metabolite profiling of fractions (Fr8-Fr10; Merge: Fr1-10) of Pacific oyster (*Crassostrea gigas*) in negative mode P10

Figure S6: MS/MS spectra of the compound **2** acquired on an Orbitrap mass spectrometer. MS/MS spectra of **2** showing the prominent product ion at m/z 240 and proposed mass fragmentation pathway of compound **2**. P11

Figure S7. ^{1}H NMR and ^{13}C NMR spectrum of methyl ester tryptophan (**10**) in $CDCl_3$ P12

Figure S8. ^{1}H NMR , ^{13}C NMR and Dept 135 spectrum of compound **3** in $CDCl_3$ P13

Figure S9. ^{1}H NMR , ^{13}C NMR and Dept 135 spectrum of compound **1** in $CDCl_3$ P14

Figure S10. ^{1}H NMR , spectra of Flazin (**4**) in $CDCl_3$ P15

Figure S11. ^{1}H NMR , spectra of Flazin (**2**) in DMSO P16

Table of contents:

Table S1. HREIMS Data of **1-8** in positive mode P17

Figure S12. A. The capture of the live imaging of lipid droplet accumulation induced by oleic acid on Hepg2 cells at different intervals of time 6h and lipid droplet staining by Oil red. B. cell viability and morphology stain by AO/EB P18

2. Supporting Information Part 1-2

S-Picture A1 Control (–OA): HepG2 cell loaded without oleic acid P20

S-Picture A2 Control (+OA)): HepG2 cell loaded with oleic acid P21

S-Picture A3 Treated flazin (**4**) HepG2 cell loaded with oleic acid treated with flazin (**4**) P22

Table S2. List of primers P23

Supporting Information Part 1-1

1. Material and Methods

1.1 Synthetic Procedure.

Synthesis of Compound **10**. Stirring solution of tryptophan **9** (500 mg, 2.45 mmol) in 10 ml of dry MeOH, $SOCl_2$ (0.8754 ml, 30 mmol) was added within 10 min dropwise under ice-cooling. Stirred the mixture for 3 h, the solvent was removed under reduced pressure, and H_2O (25 ml) was added. Then the solution was extracted with AcOEt after adjusted to 9–10 with aq. NaOH solution. The organic layer was washed with brine, filtered, and evaporated to give the compound **10** (^{1}H and ^{13}C, see Figures S7). Synthesis of Compounds **1** and **3.** To a stirred soln. Compound **10** (218 mg, 1.00 mmol), 5-Acetoxymethylfurfural (**1**, 168 mg, 1.00 mmol) and Nano CuO (7.955 mg, 0.1 mmol) were added 6 ml of DMF. The mixture was stirred at 90°C for 16 h. The pH of the aqueous solution was adjusted to 9–10 with aq NaOH solution after adding H_2O. After extracting the solution with AcOEt, the organic layer was washed with brine, filtered, and evaporation under reduced pressure to give compound **3** with compound **1** (^{1}H and ^{13}C, see Figures S8 and S9, Table S1).The aromatic amino acid tryptophan (**9**) and compound **12** were subjected to acid conversion at 90 °C in acetic acid to obtain compound **2** (^{1}H and ^{13}C, see in Supporting Information).
Synthesis of Compound **4**. To a stirred soln. compound **3** in MeOH was added with NaOH (2 mol/L). Compound **4** was obtained after stirring the mixture at 65°C for 5 h. (^{1}H and ^{13}C, see Figures S10 and S11). We also prepared similar compounds without any substituent at the C-1 position in the C-ring using a one-pot Pictet–Spengler reaction (**6**–**8**).

Synthetic β-carboline alkaloids (**1**–**8**):
CAS Registry Number of all the series of synthetic β-carboline alkaloids (1–8) were subjected to biological evaluation: 1*H*-Pyrido[3,4-*b*]indole-3-carboxylic acid, 1-[5-[(acetyloxy)methyl]-2-furanyl]-2,3,4,9-tetrahydro-, methyl ester, (1*S*-*trans*)-(9CI) (**1**, CAS Registry Number 115107-75-0; 115107-76-1); 2,3,4,9-Tetrahydro-1-[5-(hydroxymethyl)-2-furanyl]-1H-pyrido[3,4-b]indole-3-carboxylic acid (**2**, CAS Registry Number 896466-77-6); flazine methyl ester acetate (**3**, CAS Registry Number 104537-95-3); flazin (**4**, CAS Registry Number 100041-05-2); (±)-Methyl 1,2,3,4-tetrahydro-β-carboline-3-carboxylate (**5**, CAS Registry Number 16253-64-8); 1,2,3,4-Tetrahydro-β-carboline-3-carboxylic acid (**6**, CAS Registry Number 6052-68-2); methyl β-carboline-3-carboxylate (**7**, CAS Registry Number 6052-68-2); and β-Carboline-3-carboxylic acid (**8**, CAS Registry Number 74214-63-4).

1.2. Cytotoxicity assay

HepG2 cells (1.5 × 10^4/well) in DMEM supplemented with 10% FBS were seeded into a 96-well plate. The cytotoxicity assay was done using CCK-8 (Dojindo Molecular Technologies) according to the manufacturer's protocol. Cell viability was determined using the CCK-8 assay (Dojindo Molecular Technologies, Rockville, USA) according to the manufacturer’s instructions. Cell suspension (100 µl/well) was inoculated in a 96-well plate (Iwaki, Japan), and once monolayers were observed, treatments were added (n = 3 per each treatment). Cells were then pre-incubated with the treatments for 24 h before assay to determine cell viability. Absorbance was measured at 450 nm using a plate reader (PerkinElmer-ARVO-MX-ID 10533234, Japan).

1.3. Lipid droplet accumulation inhibition assay and Real-Time LDAI accumulation

Lipid droplet accumulation inhibition (LDAI) activity was determined using an oil red assay in a 24-well plate according to the manufacturer’s instructions (n = 4 per each treatment). Real-time oleic induced-LD inhibition was performed by using a Nikkon microscope. Morphological analysis of HepG2 cells after treatment with control(-OA) and (+OA) or compound at different concentrations was determined by staining with AO/EB.

1.4. Oil Red O Staining.

HepG2 cells were cultured and treated in 35 mm dishes, as described above. Oil Red O dye was used for LD staining. Oil Red O was dissolved in isopropanol as 3 mg/mL and diluted with water so that isopropanol became 60%. Oil Red O solution was filtered by a 0.4 µm pore syringe filter. For cellular staining, the culture medium was removed from each dish, and cells were washed and rinsed with 1× PBS. Cells were then fixed for 20 min in 10% formalin and washed twice using PBS. Then, Oil Red O dye was added to the dishes and incubated for 20 min. Finally, the dye was removed from each dish and cells

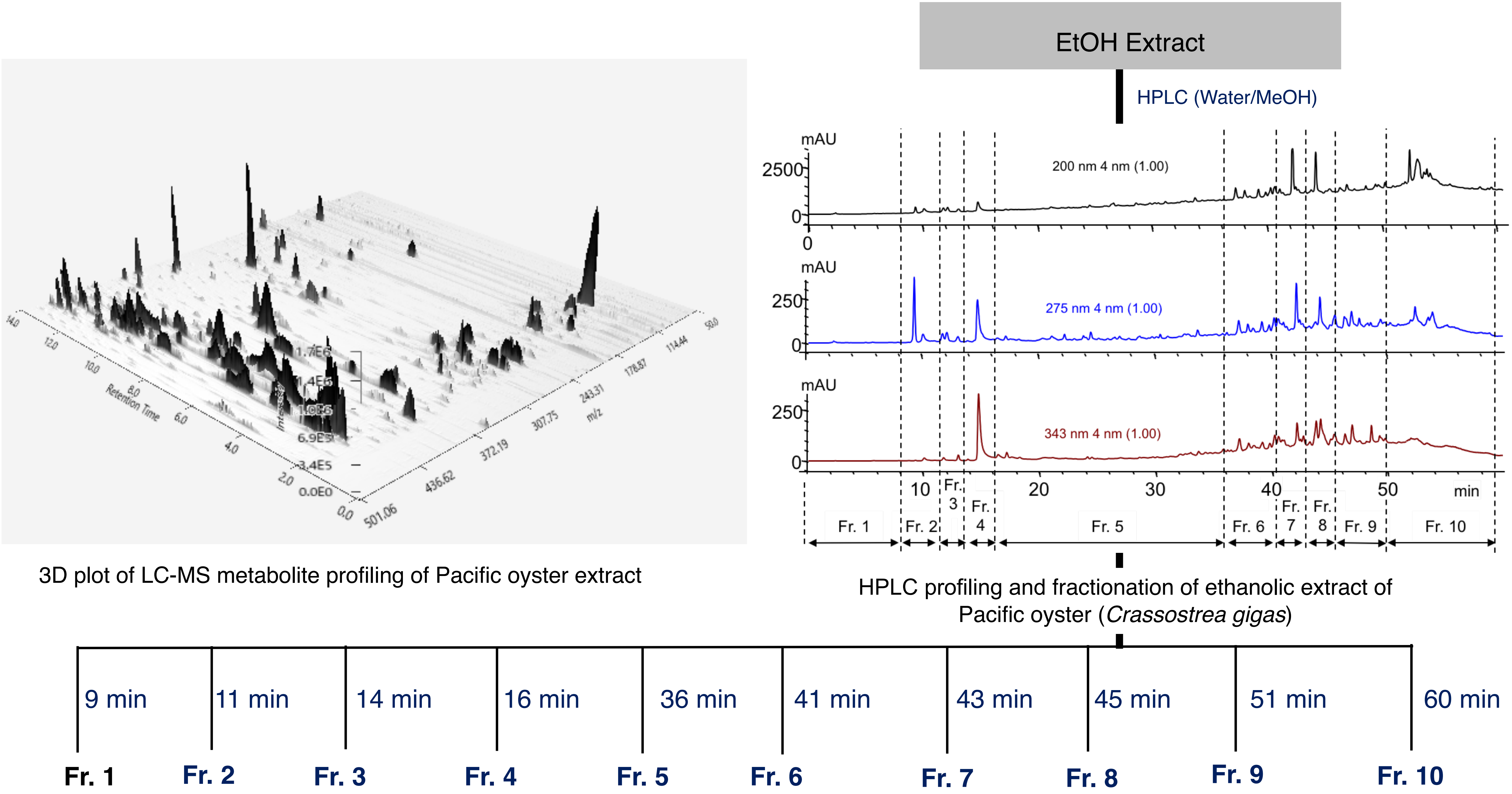

Figure S1: Fractionation of ethanolic extract of Pacific oyster (*Crassostrea gigas*)

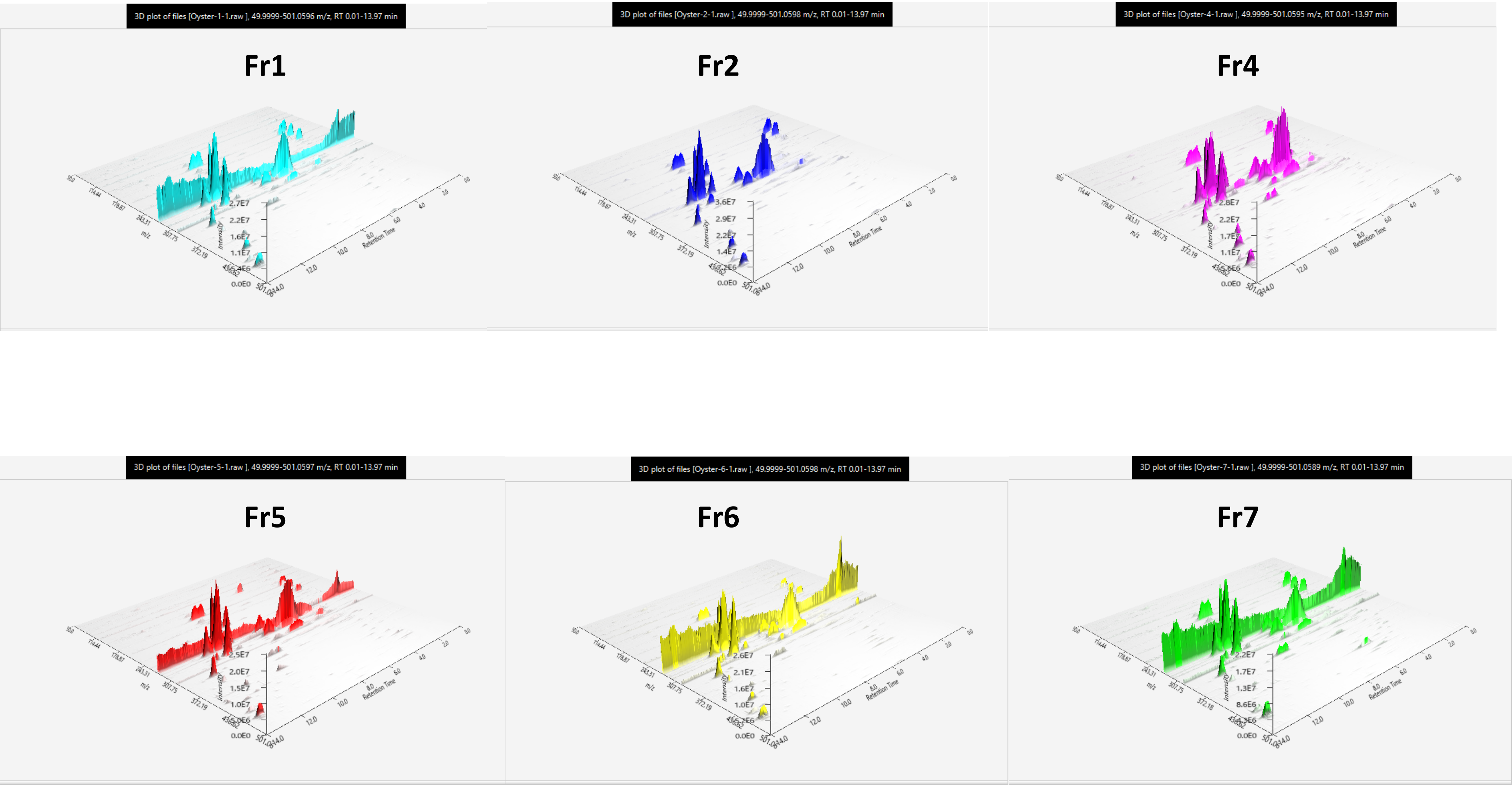

Figure S2: 3D plot of LC-MS metabolite profiling of Pacific oyster fractions (Fr1,Fr2, Fr4-Fr7) fractions in positive mode

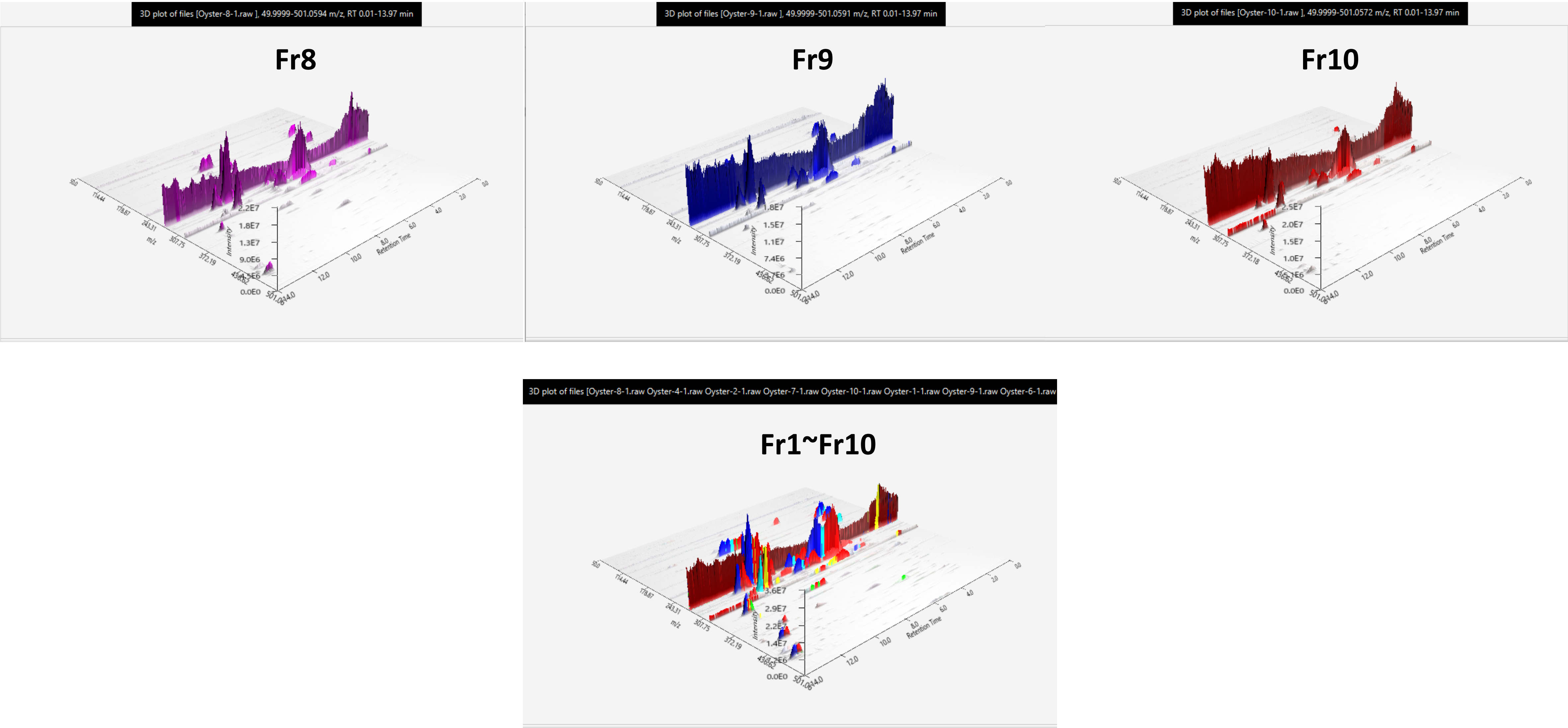

Figure S3: 3D plot of LC-MS metabolite profiling of fractions (Fr8-Fr10; Merge: Fr1-10) of Pacific oyster (*Crassostrea gigas*) in positive mode

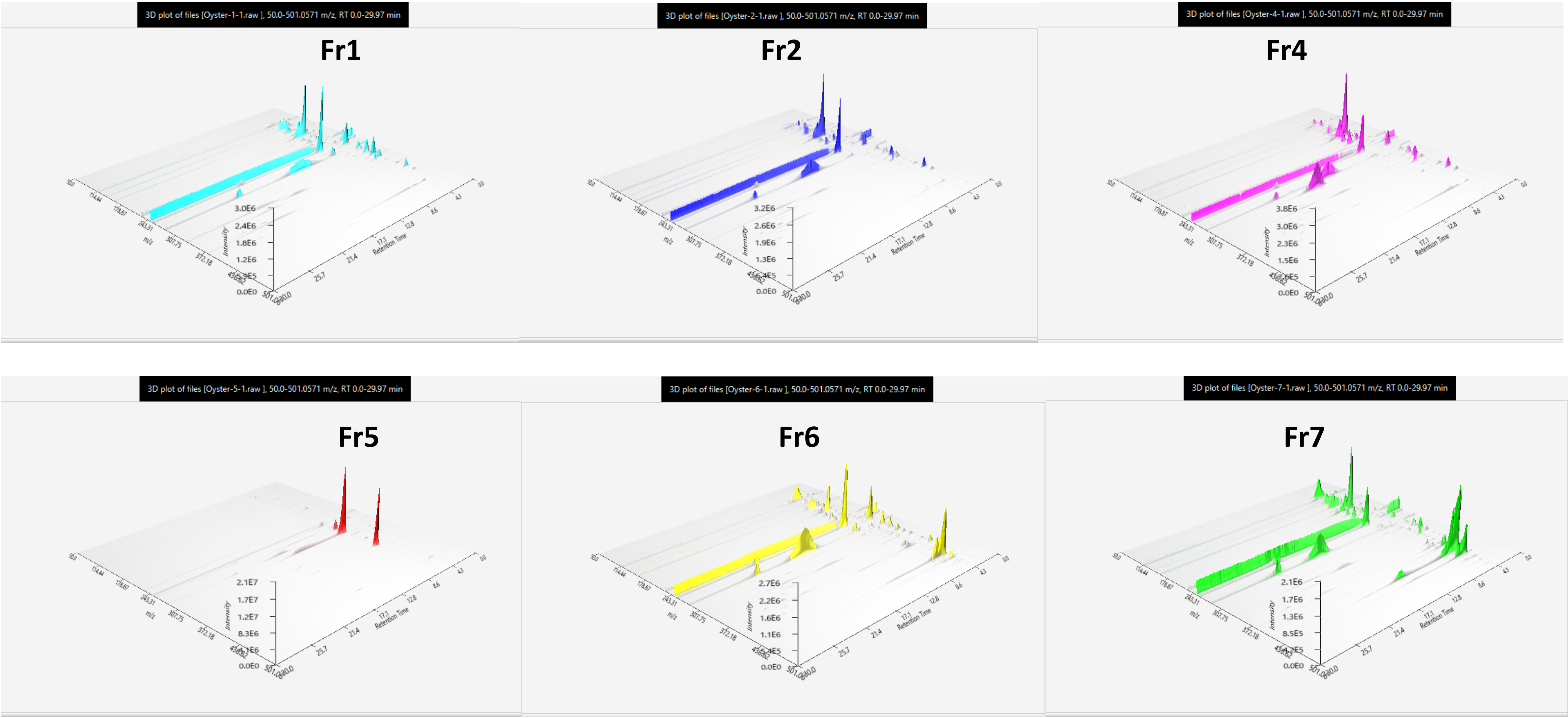

Figure S4: 3D plot of LC-MS metabolite profiling of Pacific oyster fractions (Fr1,Fr2, Fr4-Fr7) fractions in negative mode

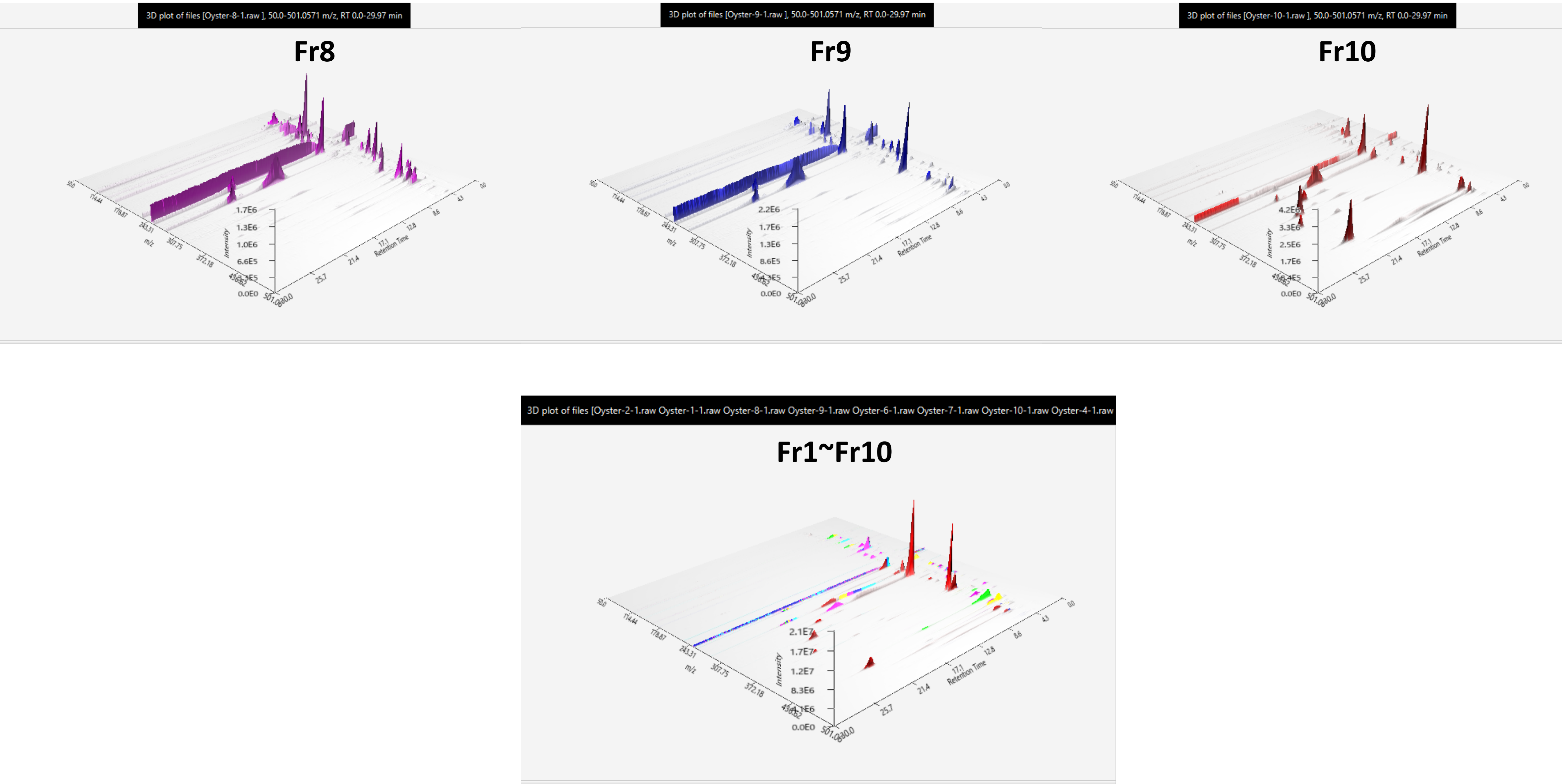

Figure S5: 3D plot of LC-MS metabolite profiling of fractions (Fr8-Fr10; Merge: Fr1-10) of Pacific oyster (*Crassostrea gigas*) in negative mode

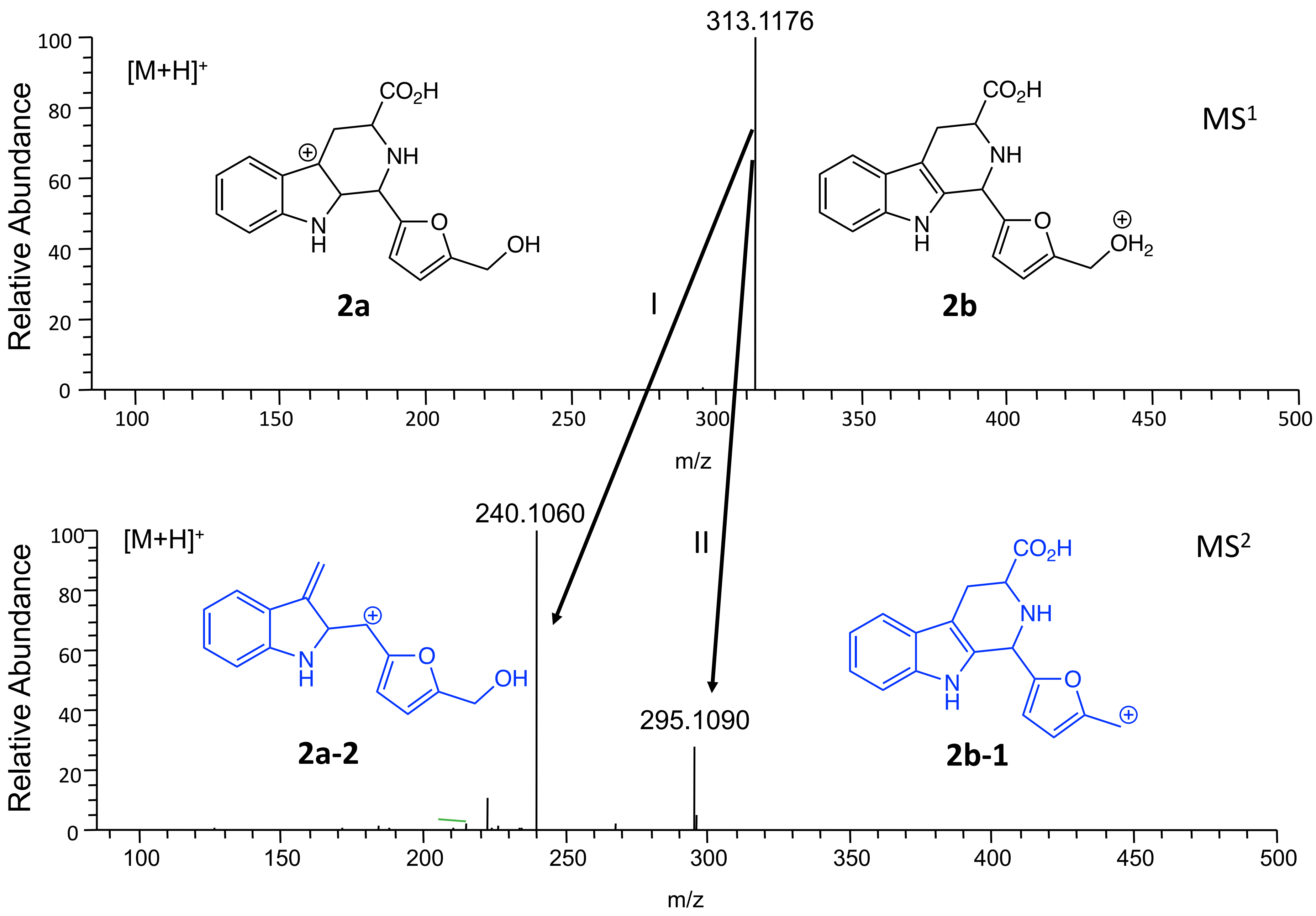

Figure S6: Proposed mass fragmentation pathway of compound **2**.

Preparation of ester L-Tryptophan derivatives

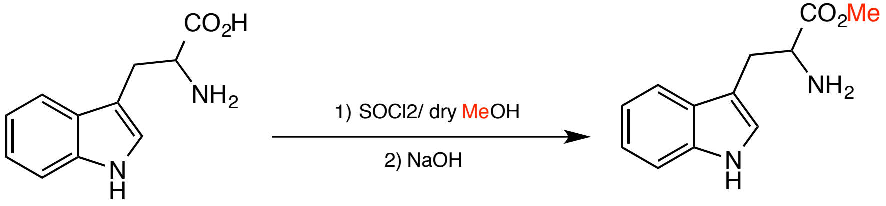

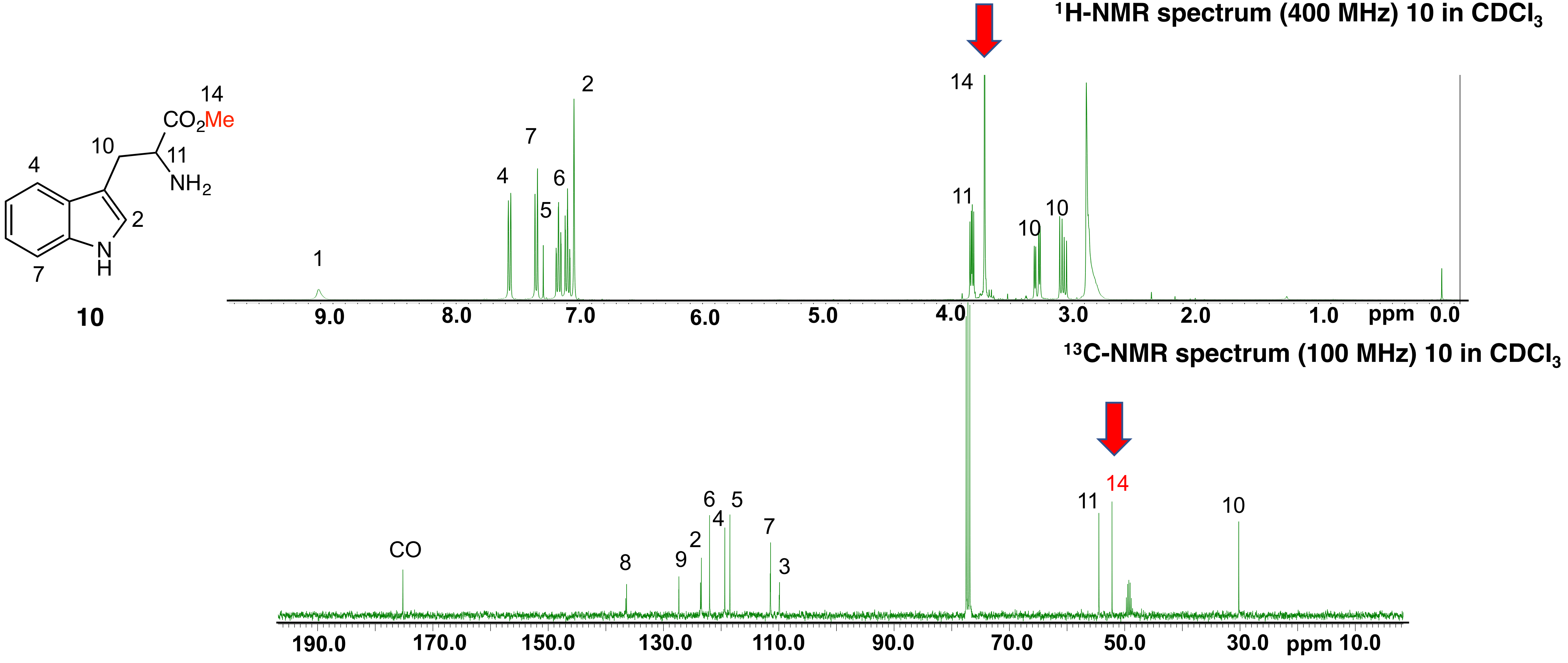

Figure S7. ^{1}H NMR and ^{13}C NMR spectrum of methyl ester tryptophan (**10**) in $CDCl_3$

Compound 3

^{1}H-NMR spectrum (400 MHz) of 3 in $CDCl_3$

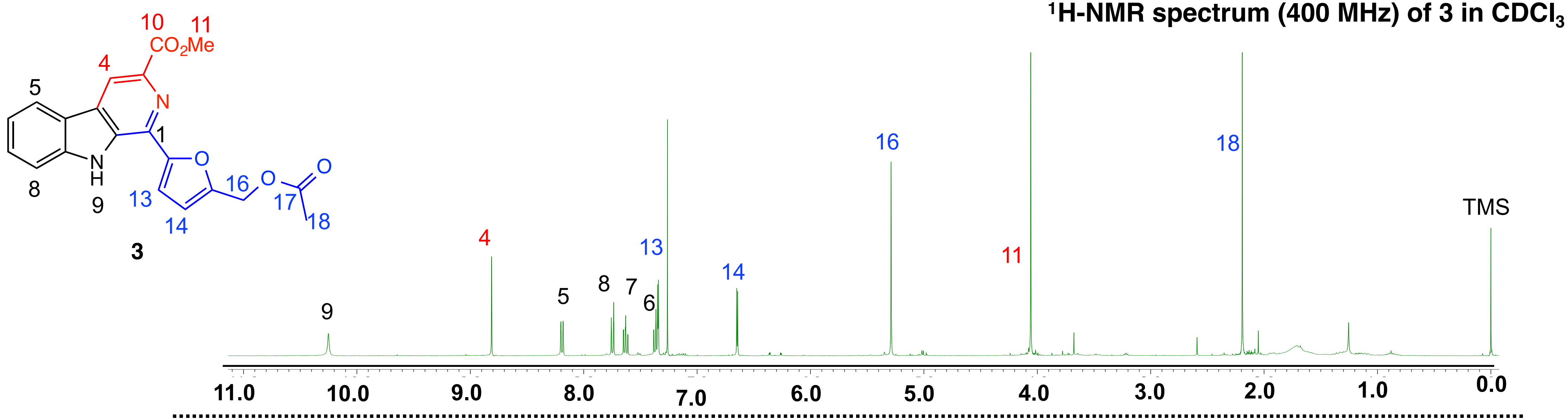

^{13}C-NMR spectrum (100 MHz) of 3 in $CDCl_3$

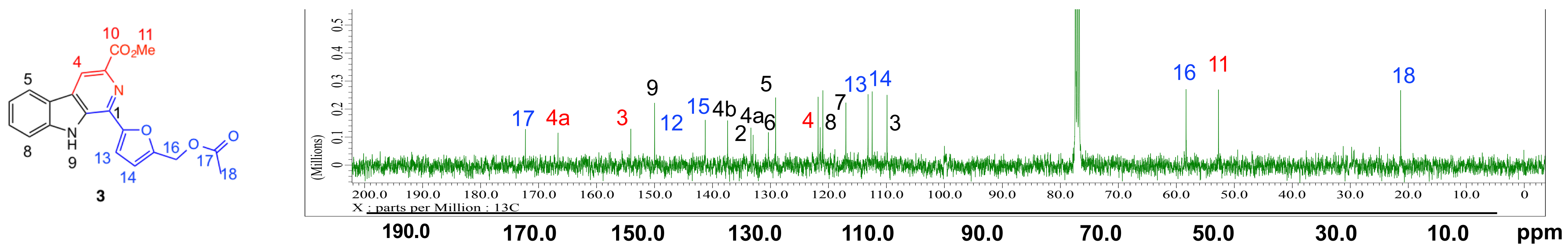

Figure S8. ^{1}H NMR and ^{13}C NMR spectrum of compound **3** in $CDCl_3$

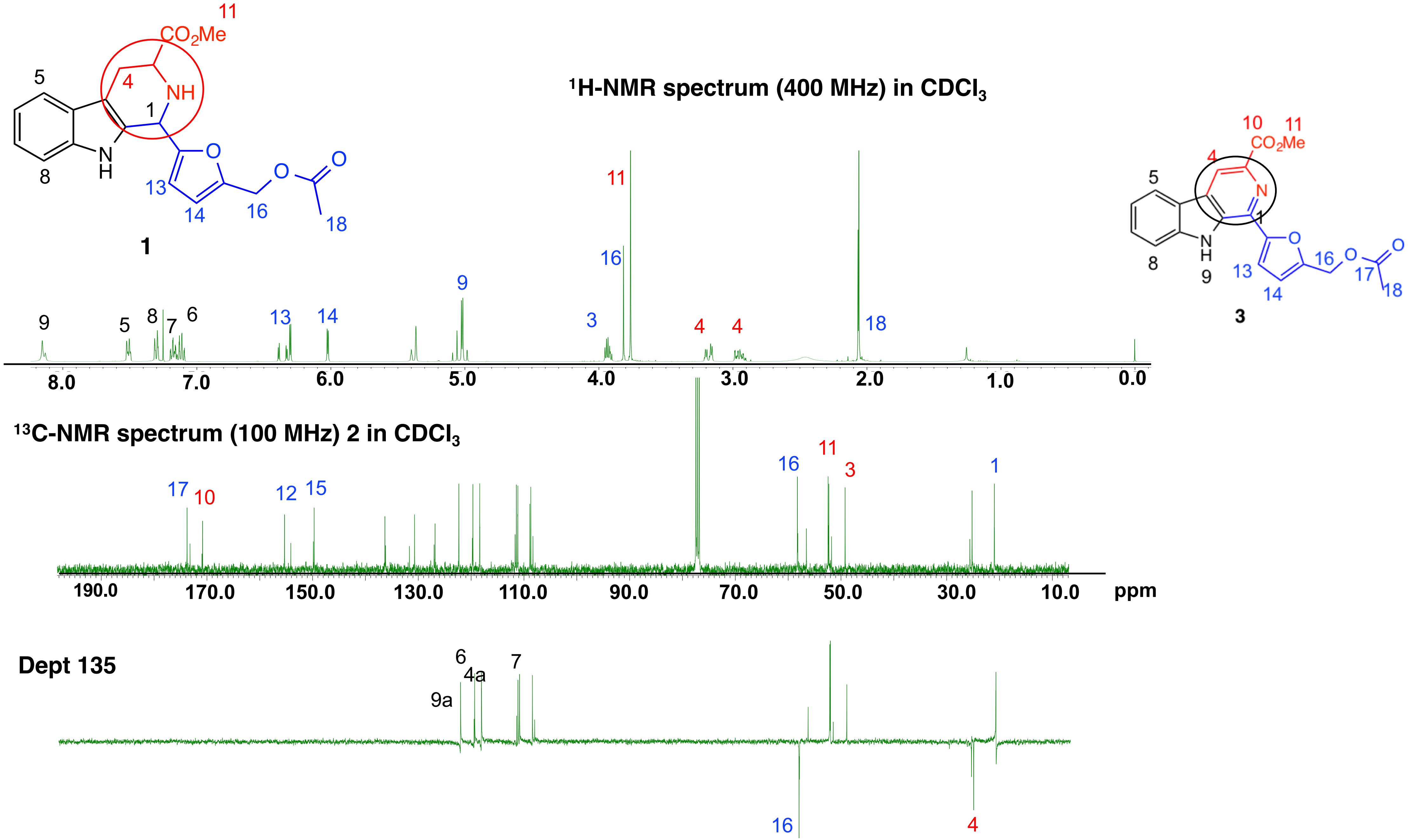

Figure S9. ^{1}H NMR , ^{13}C NMR and Dept 135 spectrum of compound **1** in $CDCl_3$

β-carboline Alkaloids

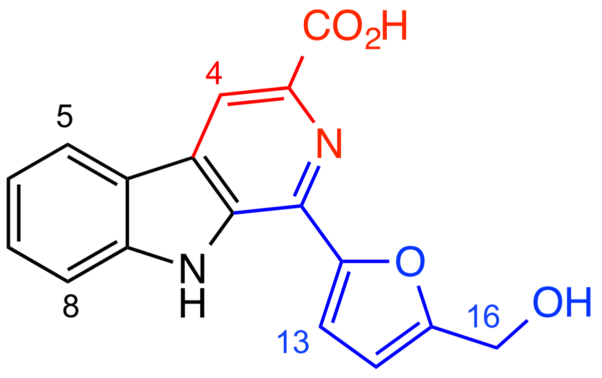

4

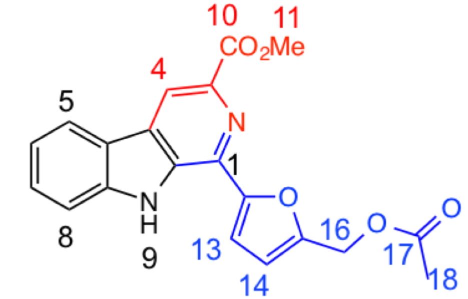

3

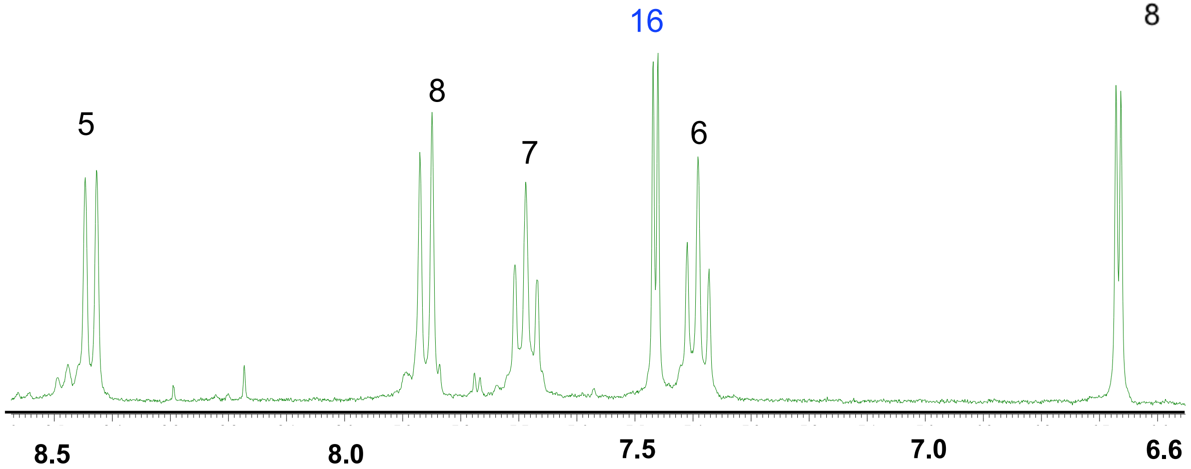

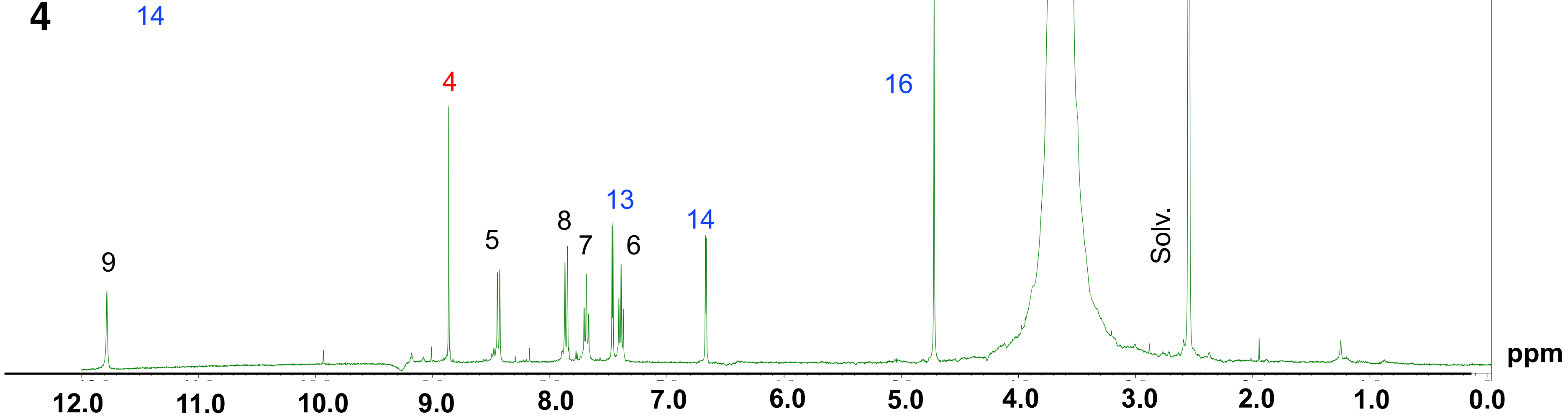

Figure S10. ^{1}H NMR , spectra of Flazin (**4**) in DMSO

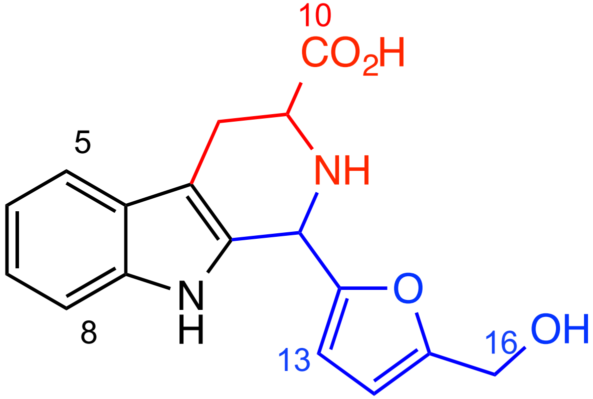

2

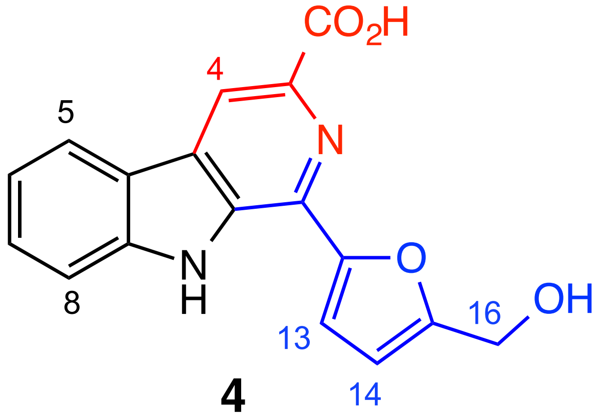

4

^{1}H-NMR spectrum (400 MHz) 2 in DMSO

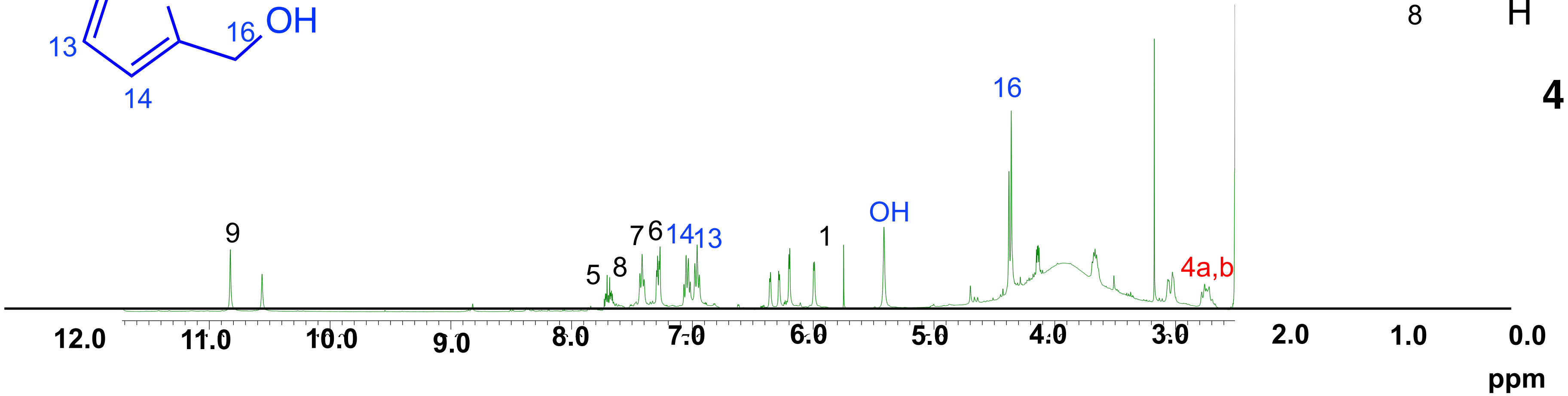

^{13}C-NMR spectrum (100 MHz) 2 in DMSO

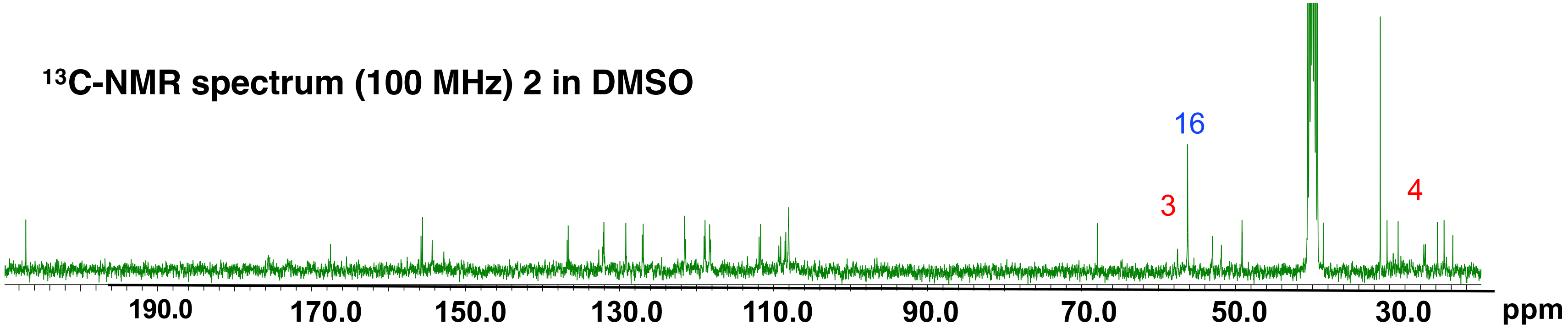

Figure S11. ^{1}H NMR and ^{13}C NMR spectrum of compound **2** in DMSO

Table S1. HREIMS Data of **1-8** in positive mode

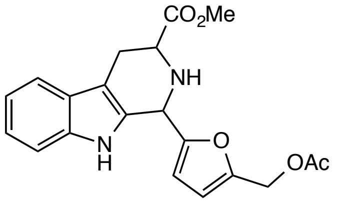

1

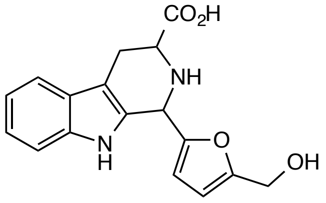

2

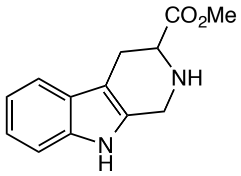

5

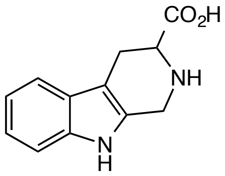

6

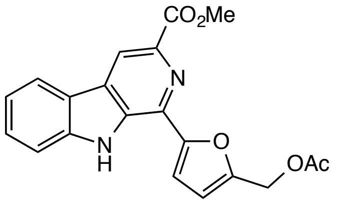

3

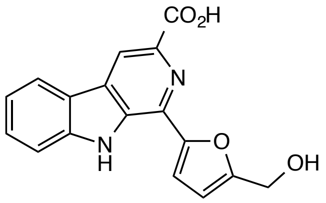

4

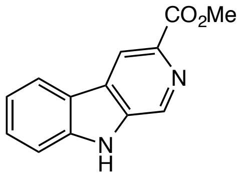

7

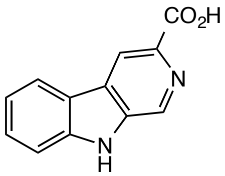

8

Compound	Ion species	Formula	Calc m/z	Experimental m/z	(Error) ppm
**1**	$[M+H]^+$	$C_{20}H_{21}N_2O_5^+$	369.1450	369.1433	-4.6
**2**	$[M+H]^+$	$C_{17}H_{17}N_2O_4^+$	313.1188	313.1176	-3.1
**3**	$[M+H]^+$	$C_{20}H_{17}N_2O_5^+$	365.1137	365.1117	-5.4
**4**	$[M+H]^+$	$C_{17}H_{13}N_2O_4^+$	309.0821	309.0801	-6.4
**5**	$[M+H]^+$	$C_{13}H_{15}N_2O_2^+$	231.1134	231.1120	-6.0
**6**	$[M+H]^+$	$C_{20}H_{13}N_2O_2^+$	217.0977	217.0968	-4.1
**7**	$[M+H]^+$	$C_{13}H_{11}N_2O_2^+$	227.0821	227.0784	-6.2
**8**	$[M+H]^+$	$C_{12}H_9N_2O_2^+$	213.0664	213.0656	-3.7

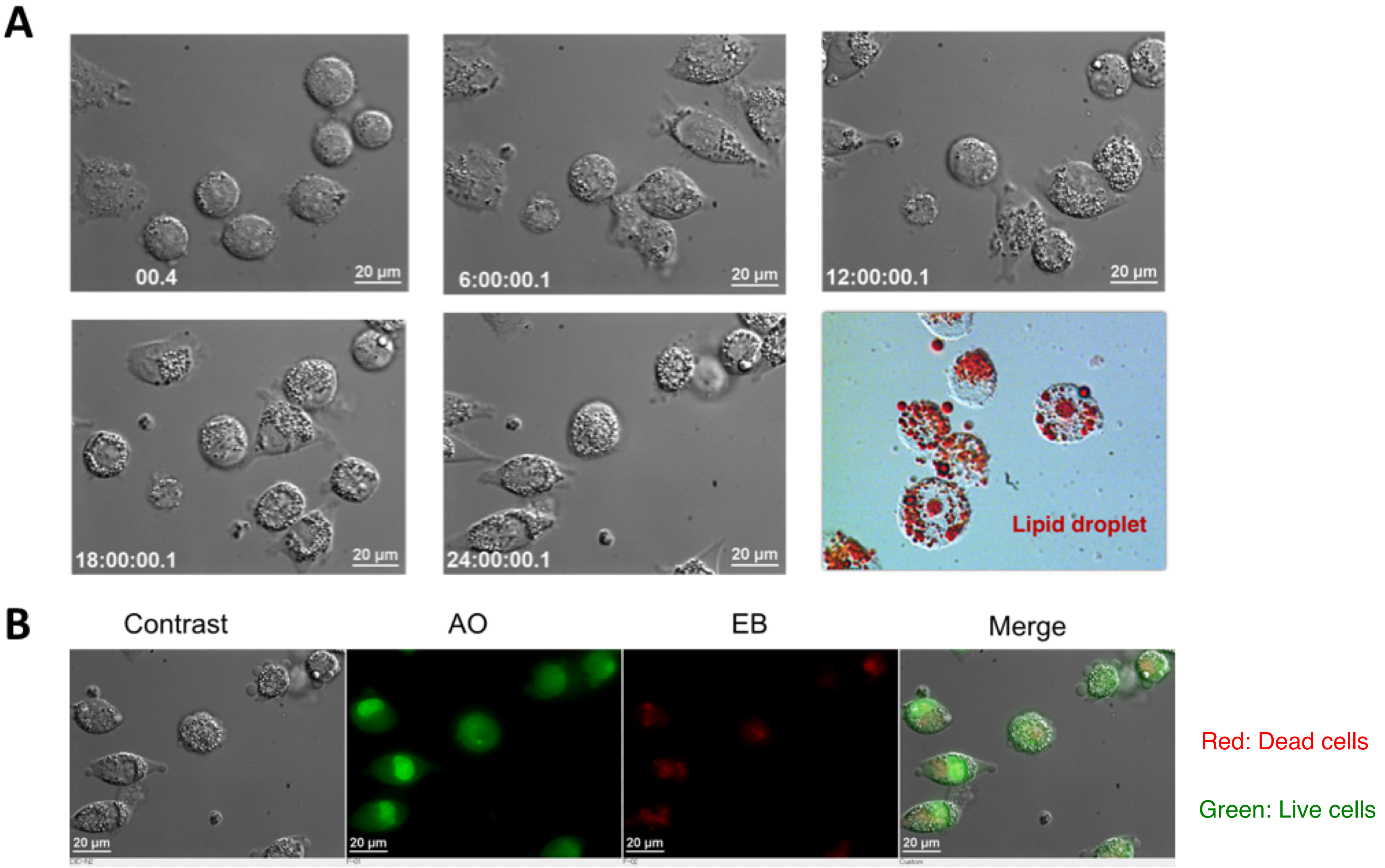

Figure S12. A. The capture of the live imaging of lipid droplet accumulation induced by oleic acid on HepG2 cells at different intervals of time 6h and lipid droplet staining by Oil red. B. cell viability and morphology stain by AO/EB

Supporting Information Part 1-2

Figure S13. S-Picture A1 Control (–OA)

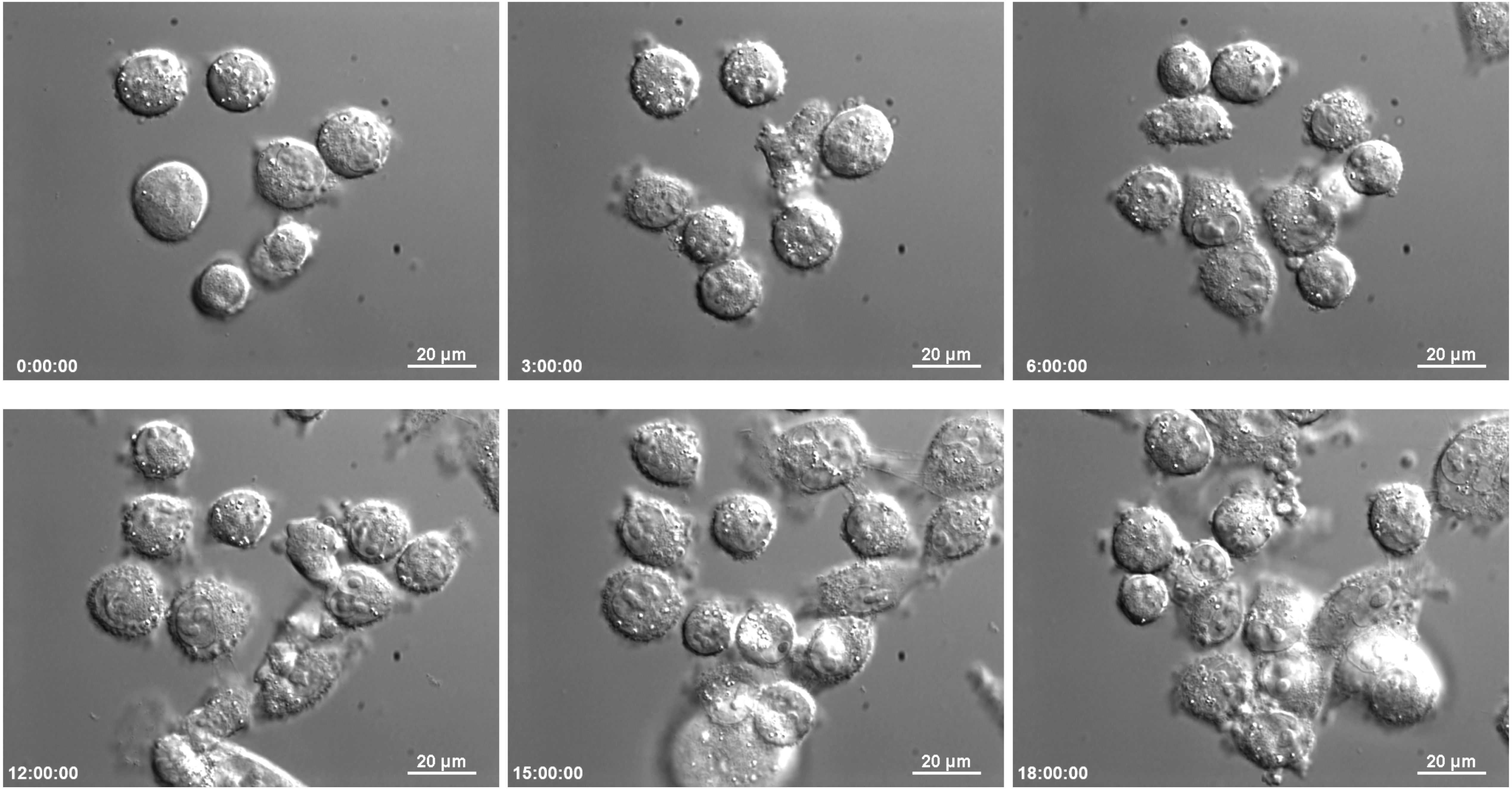

Figure S14. S-Picture A2 Control (+OA)

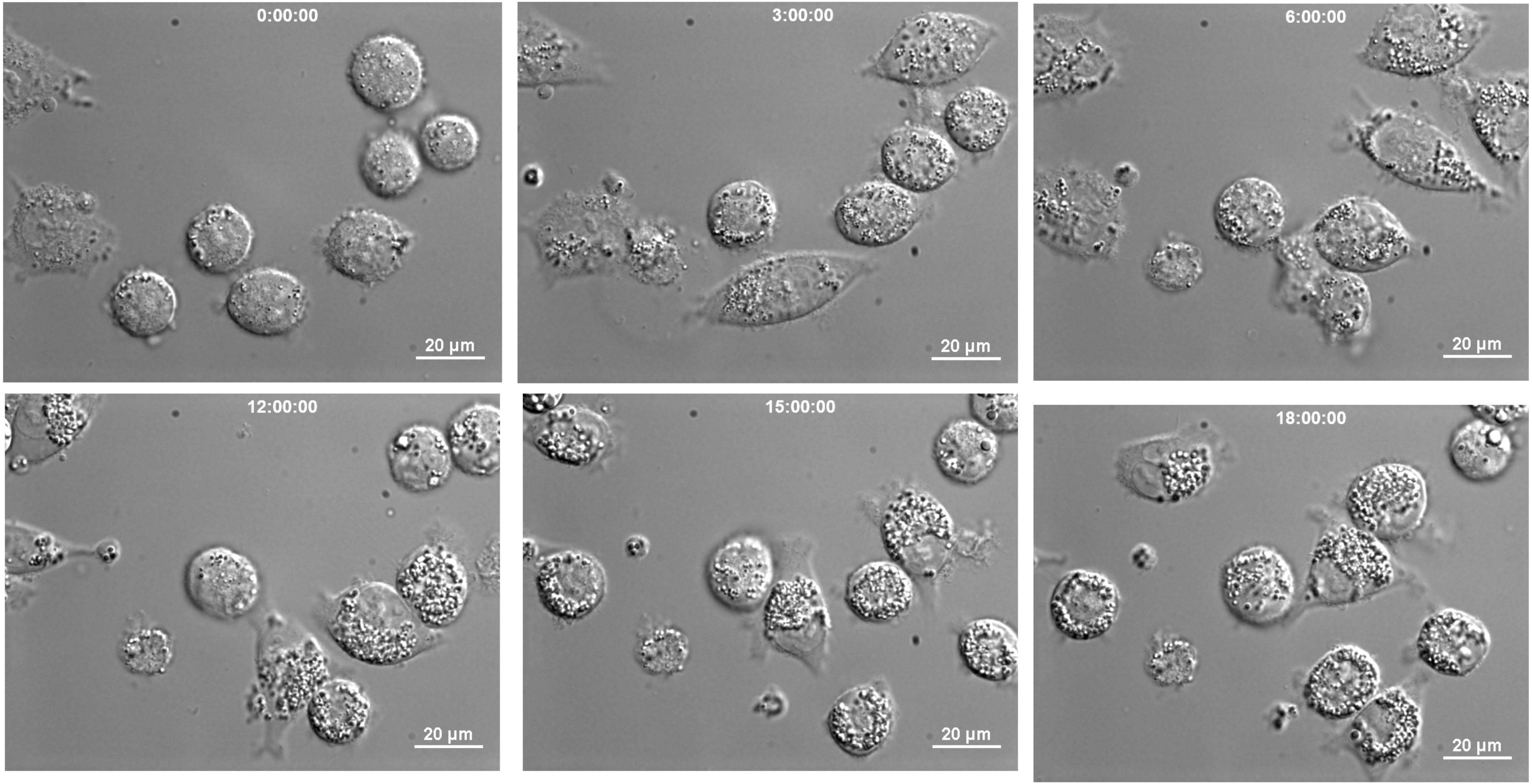

Figure S15. S-Picture A3 Treated (**4**)

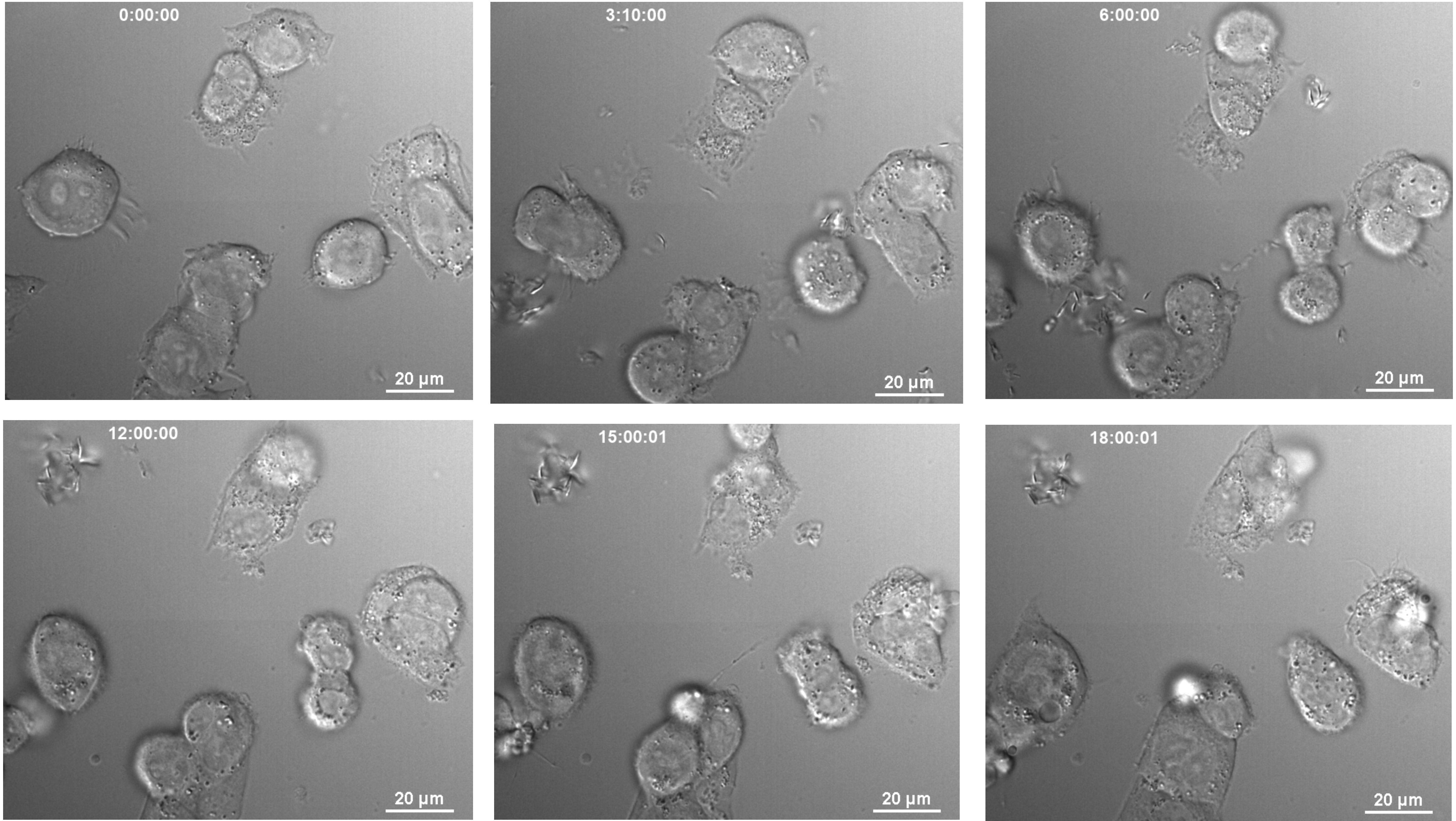

Table S2. List of primers

Gene		Primer sequences (5' → 3')			references:
ATGL	F:	ACCAGCATCCAGTTCAACCT	R:	ATCCCTGCTTGCACATCTCT	Adipose triglyceride lipase expression in human adipose tissue and muscle. Role in insulin resistance and response to training and pioglitazone
DGAT1	F:	TATTGCGGCCAATGTCTTTGC	R:	CACTGGAGTGATAGACTCAACCA	https://www.hindawi.com/journals/omcl/2018/8515343/tab1/
SREBP1	F:	CAGCCCACTTCATCAAGG	R:	ACTGTTGCCAAGATGGTTCCG	https://www.hindawi.com/journals/omcl/2018/8515343/tab1/
FASN	F:	AACTCCTGCAAGTTCTCCGA	R:	GCTCCAGCCTCGCTCTC	https://www.hindawi.com/journals/omcl/2018/8515343/tab1/
SCD1	F:	GACGATGAGCTCCTGCTGTT	R:	CTCTGCTACACTTGGGAGCC	https://www.hindawi.com/journals/omcl/2018/8515343/tab1/
GAPDH	F:	GAAGGTGAAGGTCGGAGTC	R:	GAAGATGGTGATGGGATTTC	Bhullar, K.S., Shang, N., Kerek, E. et al. Mitofusion is required for MOTS-c induced GLUT4 translocation. Sci Rep 11, 14291 (2021). https://www.nature.com/articles/s41598-021-93735-2
